# Low‐Viscosity Concentrated Lithium Chloride Solution with Unsymmetrical Ditopic Receptors in Organic Solvents

**DOI:** 10.1002/cphc.202500601

**Published:** 2025-10-26

**Authors:** Tsubasa Mimuro, Reo Sugawara, Manabu Hirasawa, Shin‐ichi Kondo

**Affiliations:** ^1^ Department of Chemistry Faculty of Science Yamagata University 1‐4‐12 Kojirakawa‐machi Yamagata 990‐8560 Japan; ^2^ Institute for Advanced Integrated Technology Resonac Co. 48 Wadai Tsukuba Ibaraki 300‐4247 Japan

**Keywords:** ditopic receptors, highly concentrated solutions, lithium chloride, solid–liquid extraction, unsymmetric structures

## Abstract

Ditopic receptors with different terminal substituents on urea moieties linked with ether groups are prepared, and the solubilization of high‐concentration LiCl by the receptors in acetonitrile is evaluated. The unsymmetrical receptors have higher solubility than the corresponding symmetrical receptors, and the complex solution of the receptor and LiCl shows reduced viscosity resulting in improved ionic conductivity, as well as suppression of solidification. In addition, selective solid–liquid extraction of LiCl by the receptors is demonstrated by the ^1^H NMR spectra compared to other alkaline and alkali earth chlorides. Density functional theory calculations suggest that this selectivity is due to the shape complementarity of the cation recognition site and the cooperative anion recognition by the two urea groups. These properties suggest that the unsymmetrical ditopic receptors can be applied to an electrolyte and LiCl extractant.

## Graphical abstract

A low viscosity and highly concentrated solution of LiCl can be prepared using ditopic receptors bearing different terminal substituents on urea moieties linked by ether groups in organic solvents. The solution shows improved ionic conductivity due to the low viscosity, as well as suppression of solidification compared to solutions with symmetrical receptors.

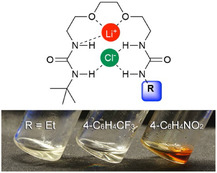

## 1 Introduction

In recent years, electric vehicles that emit less CO_2_ have been developed and popularized for the progress of a carbon‐neutral society. In these fields, lithium‐ion batteries (LIBs) are attracting attention because of their light weight and high battery capacity, and they are being applied in a wide range of applications from portable power sources such as smartphones to fixed power sources such as stationary storage batteries.^[^ ^1^ ^]^ Until now, LiPF_6_ has often been used as the electrolyte in LIBs,^[^ ^2^ ^]^ but this salt is expensive, which prevents the reduction of the cost of batteries. In addition, PF_6_^−^ is known to be easily hydrolyzed by moisture to produce hydrogen fluoride, which degrades battery performance and has a negative impact on the environment when disposed of as waste.^[^ ^3^ ^]^ Therefore, there is a need for a cheap and moisture‐stable lithium salt to replace LiPF_6_. Moreover, lithium salts are derived from brine and mineral deposits. Despite the sustainability of these deposits, concerns have been raised regarding the environmental impact if the lithium supply continues to meet the demand of recent years.^[^ ^4^, ^5^, ^6^, ^7^ ^]^ Notwithstanding these challenges, the recovery of lithium remains below 1% on a global scale. This underscores the necessity for the development of newer recovery technologies for various lithium resources, including waste batteries, seawater, and brine, to meet future demands.^[^ ^8^ ^]^

A heteroditopic receptor bearing two different recognition sites in one molecule has attracted attention to solve such problems. In particular, an ion pair receptor, which can simultaneously recognize anion and cation species, encapsulates the guest salt to increase the solubility of the complex in organic solvents.^[^ ^9^, ^10^ ^]^ In addition, the ion‐pair receptor exhibits relatively high recognition ability and selectivity for a specific salt due to the contribution of electrostatic interactions between the two ions.^[^ ^11^, ^12^ ^]^ Then, the heteroditopic ion‐pair receptor is widely applied to salt solubilization, extraction, and membrane transport.^[^ ^13^, ^14^ ^]^ Sessler et al. reported the solid–liquid extraction of LiCl from a mixture of NaCl and KCl using a heteroditopic receptor based on calix[4]pyrrole, which was very effective as a LiCl‐selective receptor; however, the multistep synthesis of the receptors and the competing salts lead to difficulties in practical applications.^[^ ^15^ ^]^ Ghosh et al. reported the selective solid–liquid extraction of KBr by an ion‐pair receptor bearing crown ether, urea, and amide groups as recognition sites,^[^ ^16^ ^]^ and Costero et al. reported solubilization of the zwitterionic form of amino acids in organic solvents by a receptor bearing crown ether and thiourea moieties;^[^ ^17^ ^]^ however, the solubilization studies were monitored only with NMR concentrations, which are lower than the practical concentration of salts such as an electrolyte in batteries. Gomez‐Vega and Lara et al. reported that a bisurea connecting with an oligoether chain showed ion‐pair recognition of alkali acetate salts.^[^ ^18^ ^]^ More recently, Romański and coworkers showed solid– and liquid–liquid extraction of LiCl by a squaramide–crown ether‐based receptor and polymer.^[^ ^19^ ^]^

We have recently reported heteroditopic receptors **1a**, **1d**, and **1f** with high solubility and selectivity for LiCl.^[^ ^20^, ^21^ ^]^ These receptors have an ether moiety as the cation recognition site and two urea groups as the anion recognition site cooperatively. These form a 1:1 complex with LiCl complex in organic solvents such as MeCN and CHCl_3_. In particular, receptor **1a** bearing terminal *t*‐butyl groups successfully solubilizes LiCl at a high concentration up to 9.6 M in MeCN.^[^ ^21^ ^]^ This high‐concentration solution of the receptor and LiCl is similar to the solubilization of the Li salt in ionic liquids with grimes reported by Watanabe et al.^[^ ^22^ ^]^ and in deep eutectic solvents composed of urea and other substances.^[^ ^23^ ^]^ Furthermore, Density functional theory (DFT) calculations and isotope substitution neutron scattering experiments have revealed that receptor **1b** recognizes a single ion‐pair of LiCl by the coordination of ether oxygens to Li^+^ and hydrogen bonds of urea NHs to Cl^−^. In addition, a single molecule of acetonitrile coordinates Li^+^ and a contact ion pair is formed by electrostatic interaction between Li^+^ and Cl^−^.^[^ ^24^ ^]^ We call this new high‐concentration solution as “a supramolecular ionic liquid.” A 3 M **1b**·LiCl solution in acetonitrile shows an ionic conductivity of 0.128 mS cm^−1^, suggesting the possibility of using LiCl as a new electrolyte that has not been used before.^[^ ^21^, ^25^ ^]^ In addition, the solubilization of other related salts, such as NaCl or KCl by **1** is not observed, indicating high selectivity for LiCl. Therefore, this selective solubilization of LiCl by receptors **1** should be applied as a new recovery technique from LiCl‐containing resources.^[^ ^26^ ^]^

**Scheme 1:**
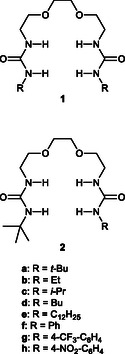
Structures of receptors **1** and **2**.

However, there are three major issues with the **1**·LiCl concentrated solutions. The first is solidification after standing of the complex solution for several days. The solid formed is amorphous rather than crystals. The formation of such solids from an electrolyte is known to show significant difficulties on separator damage and battery performance degradation;^[^ ^27^ ^]^ therefore, such solidification is a major drawback for battery applications. The second is the high viscosity of the solution, which is generally known to lead to a decrease in the transport rate of Li^+^ and the ionic conductivity in the electrolyte. The third is the competition for the solid–liquid extraction of LiCl. Receptors **1** also extract relatively low concentration of MgCl_2_, which is the main content in bitterns due to their similar ionic radii;^[^ ^28^ ^]^ therefore, it is necessary to design receptors with higher LiCl selectivity. To address these issues, we designed and synthesized a series of unsymmetrical receptors **2** with different terminal substituents (**Scheme** **1**). Since receptor **1a**, which has two *t*‐butyl groups as terminal substituents, is the most soluble in organic solvents, one of the terminal residues of **2** was fixed as a *t*‐butyl group. It is well known that molecular symmetry has a significant effect on the solubility of organic compounds. Highly symmetrical molecules generally have higher melting points and lower solubility than structurally similar unsymmetrical molecules.^[^ ^29^, ^30^, ^31^, ^32^ ^]^ In addition, aromatic substituents on a urea moiety with electron‐withdrawing groups increase the acidity of the urea group, allowing strong association with an anion. However, strong hydrogen bonds also lead to intra‐ and intermolecular interactions that result in lower solubility. In other words, there is a trade‐off between ion recognition ability and solubility; however, this trade‐off should be resolved by introducing different substituents as **2**. In this study, we study the association properties with cations and anions, the complexation with LiCl with the suppression of solidification, the reduction of the viscosity, the improvement of the ionic conductivity, and the solid–liquid extraction by receptors **2**.

## 2 Results and Discussion

### 2.1 Synthesis

We have reported the synthesis and the properties of the symmetrical receptors **1a** (R = *t*‐Bu), **1d** (R = Bu), and **1f** (R = Ph). Receptor **1a** showed the highest solubility in various organic solvents, and it was also the most difficult to solidify the solution of the receptor**·**LiCl complex. Therefore, for the unsymmetrical receptor **2**, we chose *t*‐Bu group as one terminal substituent and different from *t*‐Bu was introduced into the other substituent.

**Scheme 2:**
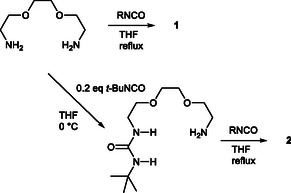
Synthesis of receptors **1** and **2**.

The unsymmetrical receptors **2** were prepared in two steps in high yield as shown in **Scheme** **2**. Bis(2‐aminoethoxy)ethane was reacted with 0.2 equiv. of *t*‐butyl isocyanate under dilute conditions in THF. The product was purified by only extraction processes. The resulting monourea **3** was reacted with different isocyanates in THF to give **2**. The corresponding receptors **1** having the identical substituents at the terminal residues were synthesized in a single step by reaction with the corresponding isocyanate from the same starting material. As shown in **Table** **1**, the melting point of receptor **2** is significantly lower than that of the corresponding symmetrical receptor **1**. It should be noted that receptor **2g** has a melting point close to room temperature. In addition, the slight decrease in the melting point of **2c**, which has structurally similar branched *t*‐butyl and isopropyl groups as terminal residues indicates the importance of the symmetry of the molecular structure on the melting point as expected. We then determined the solubility of each receptor in CHCl_3_ and MeCN. All unsymmetrical receptors had a solubility greater than 500 mM in CHCl_3_, and receptors **2b**, **2g**, and **2h** had a solubility greater than 100 mM in MeCN (Table 1). In previous reports, the low solubility of **1d** and **1f** was detrimental to the solubilization of LiCl; then, the significant improvement of the solubility of receptor **2** should have a positive effect on complex formation with LiCl.

**Table 1:** Melting point and solubility of receptors 1 and 2.

Receptor	R	M. p. [°C]	Saturated concentration [mM]	
			CHCl_3_	MeCN
**1a**	*t*‐Bu	151.0–156.0	>500	16
**1b**	Et	137.6–140.1	14.9	5.27
**1c**	*i*‐Pr	145.0–148.1	12.5	2.47
**1d**	Bu	127.5–128.5	73	1.4
**1e**	C_12_H_25_	135.5–136.0	0.79	NDa)
**1f**	Ph	130.0–130.5	11.7	4.90
**1g**	4‐CF_3_‐C_6_H_4_	148.5–150.5	15.7	33.7
**1h**	4‐NO_2_‐C_6_H_4_	170.0–171.0	1.10	5.49
**2b**	Et	77.0–79.0	>500	250
**2c**	*i*‐Pr	132.0–133.0	>500	19
**2d**	Bu	71.0–73.0	>500	71
**2e**	C_12_H_25_	65.0–67.0	>500	61
**2f**	Ph	89.0–95.5	>500	95
**2g**	4‐CF_3_‐C_6_H_4_	44.5–46.5	>500	110
**2h**	4‐NO_2_‐C_6_H_4_	51.0–53.0	>500	380

a) Not determined due to the low solubility.

### 2.2 Association Properties

**Figure 1:**
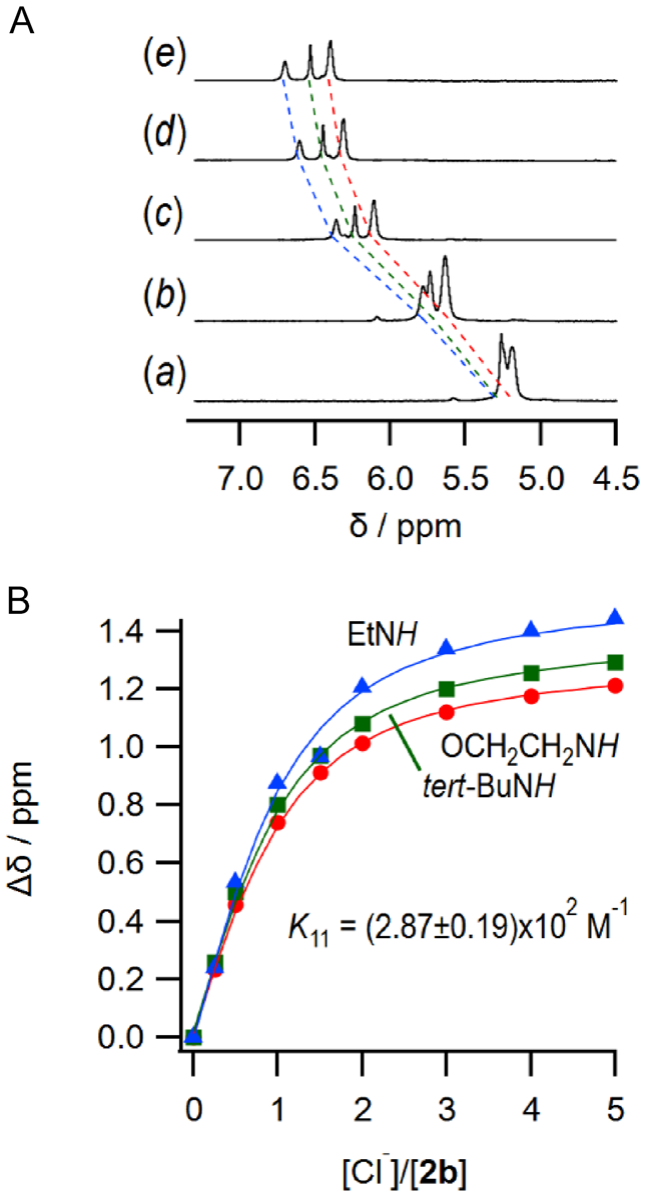
A) ^1^H NMR spectra of **2b** upon the addition of 0 (*a*), 0.5 (*b*), 1.5 (*c*), 3.0 (*d*), and 5.0 (*e*) equiv. of TBACl in MeCN‐*d*_3_ at 298 K. The blue, green, and red lines correspond to EtN*H*, *tert*‐BuN*H*, and OCH_2_CH_2_N*H* (two NHs are overlapped), respectively. B) The chemical shift changes of the urea NHs of **2b** upon the addition of TBACl. [**2b**] = 1.0 × 10^−2^ M.

The association properties of receptor **2** were then studied by means of ^1^H NMR in MeCN‐*d*_3_ and UV–vis titrations in MeCN. Since the difference between the unsymmetrical and symmetrical structures in ion recognition is thought to lie mainly in the urea moieties as the anion recognition sites, the association properties of the receptors **2** with anionic species, such as AcO^−^ and Cl^−^ as tetrabutylammonium salts, were studied in comparison with the symmetrical receptors **1**. The NH peaks of the urea moieties of **1a**, which overlapped at around 5.07 ppm, were shown to be downfield shifted and split into two peaks upon the addition of anions. In contrast, for the unsymmetrical **2b**, the NH protons appeared as two broad peaks at 5.19 and 5.26 ppm, and the peaks were similarly shown to shift downfield upon the addition of an anion and split into four peaks. This change was also observed for **2d**, suggesting a difference in the coordination structure of the anion due to the unsymmetrical structure, and fitting these changes using nonlinear least‐squares curve fitting analysis showed that the receptor and anion were associated in a 1:1 ratio (**Figure** **1**).

**Figure 2:**
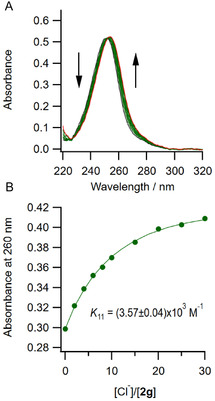
A) UV–vis spectral change of **2g** upon the addition of TBACl in MeCN at 298 K. B) Absorbance change of 2g at 260 nm upon the addition of TBACl in MeCN at 298 K. [**2g**] = 2.0 × 10^−5^ M.

Second, UV–vis titration was performed for **2f**–**h** bearing an aromatic residue at one of the terminal groups. Receptors **2f**, **2g**, and **2h** have maximum absorptions at 241, 251, and 338 nm, respectively, and the absorption maxima showed bathochromic shifts by about 4, 6, and 20 nm through an isosbestic point at 242, 253, and 343 nm, respectively. Fitting analyses of these changes by nonlinear least squares method clearly showed a 1:1 association manner (**Figure** **2**), and the results are summarized in **Table** **2**. The association constants of **2b** and **2d** with an aliphatic group at R were comparable to those of the corresponding **1a**, **1b**, and **1d**, indicating that the unsymmetrical structure of the receptors has a small contribution to the recognition capabilities. The association constants of the receptors with aromatic terminal residues (**1f**, **1g**, and **1h**) were significantly larger than those of the receptors bearing aliphatic residues due to the higher acidity of the N‐H of the urea moieties by the conjugation resulting in the formation of stronger hydrogen bonds with anions. It should be noted that receptors **1f** and **1g** containing aromatic residues with electron‐withdrawing groups showed significant association ability, but low solubilities as indicated in Table 1. Unsymmetrical receptors with an aromatic moiety at one terminal residue (receptors **2f**, **2g**, and **2h**) showed, as expected, an intermediate character between receptors with aliphatic and aromatic residues at both terminals for the association with anions. These results clearly indicate that the solubility and the association ability of the receptors are kept compatible by adopting the unsymmetrical structure of receptors **2**.

**Table 2:** The association constants of receptors 1 and 2 for anions.

Receptor	R	*K*_11_ [M^−1^]	
		TBAAcO	TBACl
**1a** a)	*t*‐Bu	(4.10 ± 0.31) × 10^2^	(1.83 ± 0.05) × 10^2^
**1b** a)	Et	(3.14 ± 0.50) × 10^2^	(1.42 ± 0.19) × 10^2^
**1d** a)	Bu	(6.31 ± 0.35) × 10^2^	(2.64 ± 0.07) × 10^2^
**1f** b)	Ph	(3.70 ± 0.04) × 10^4^	(3.44 ± 0.18) × 10^4^
**1g** b)	4‐CF_3_‐C_6_H_4_	(9.87 ± 0.25) × 10^5^	(4.56 ± 0.03) × 10^4^
**1h** b)	4‐NO_2_‐C_6_H_4_	(2.17 ± 0.08) × 10^6^	(7.12 ± 0.14) × 10^4^
**2b** a)	Et	(3.81 ± 0.34) × 10^2^	(2.87 ± 0.19) × 10^2^
**2d** a)	Bu	(5.64 ± 0.13) × 10^2^	(1.45 ± 0.07) × 10^2^
**2f** b)	Ph	(1.83 ± 0.07) × 10^4^	(2.27 ± 0.24) × 10^3^
**2g** b)	4‐CF_3_‐C_6_H_4_	(1.34 ± 0.15) × 10^4^	(3.57 ± 0.04) × 10^3^
**2h** b)	4‐NO_2_‐C_6_H_4_	(2.30 ± 0.26) × 10^5^	(5.79 ± 0.69) × 10^3^

a) Determined by ^1^H NMR titrations in MeCN‐*d*_3_ at 298 K. [Receptor] = 1.0 × 10^−2^ M.

b) Determined by UV–vis spectral titrations in MeCN at 298 K. [Receptor] = 2.0 × 10^−5^ M.

Next, ^1^H NMR titrations of **2b** and **2g** with LiPF_6_ and NaPF_6_ as guests were performed to confirm the cation binding abilities of the receptors in comparison to the structurally similar **1**. Both receptors exhibited a downfield shift of methylene protons adjacent to the ether group upon the addition of LiPF_6_ (Figure S27, Supporting Information) as observed for **1a**, and the titration data can be fitted by the least‐squares method suggesting a receptor:guest = 1:1 stoichiometry (Figure S28, Supporting Information). The association constants of **2b** and **2g** for Li^+^ (**Table** **3**) were (9.64 ± 0.28) × 10^2^ and (4.07 ± 0.14) × 10^2^ M^−1^, respectively, which were similar to those of **1a** ((2.48 ± 0.03) × 10^2^ M^−1^). In addition, the chemical shift changes of receptors **2b** and **2g** upon the addition of NaPF_6_ were smaller than those upon the addition of LiPF_6_, and the association constants of **2b** and **2g** for Na^+^ were (7.22 ± 0.94) × 10^1^ and (3.04 ± 0.02) × 10^1^ M^−1^, respectively. The selectivity for Li^+^ and Na^+^ was not significantly different, indicating that receptor **2** is also Li^+^ selective receptors.

**Table 3:** The association constants of receptors **2b** and **2g** for cations by ^1^H NMR titrations in MeCN‐*d*_3_.

Receptor	R	*K*_11_ [M^−1^]	
		LiPF_6_	NaPF_6_
**2b**	Et	(9.64 ± 0.28) × 10^2^	(7.22 ± 0.94) × 10^1^
**2g**	4‐CF_3_‐C_6_H_4_	(4.07 ± 0.14) × 10^2^	(3.04 ± 0.02) × 10^1^

### 2.3 Solubilization of LiCl

**Figure 3:**
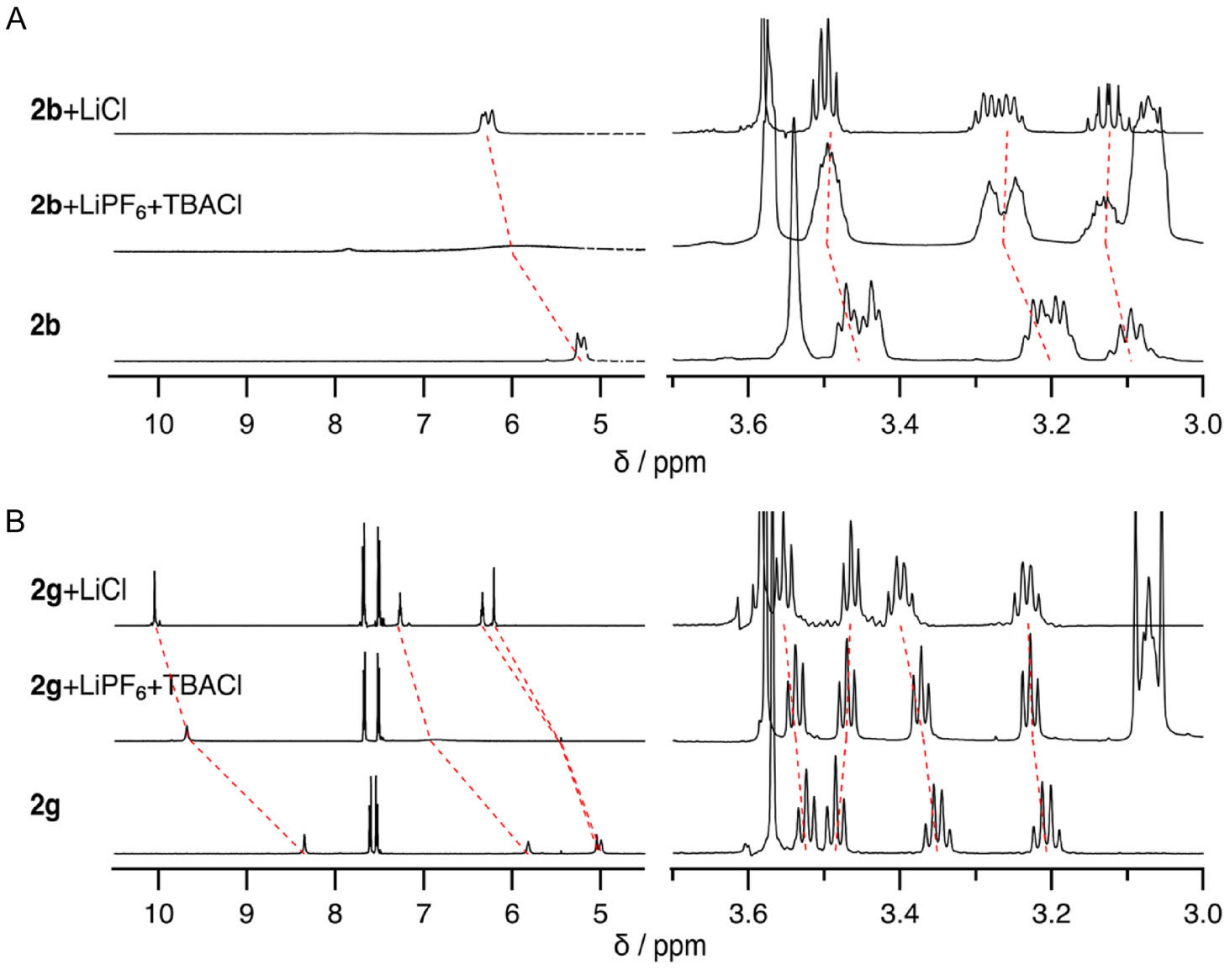
^1^H NMR spectra of receptors A) **2b** and B) **2g** in the absence and the presence of TBACl + LiPF_6_ and LiCl in MeCN‐*d*_3_ at 298 K. [Receptor] = 1.0 × 10^−2^ M.

**Figure 4:**
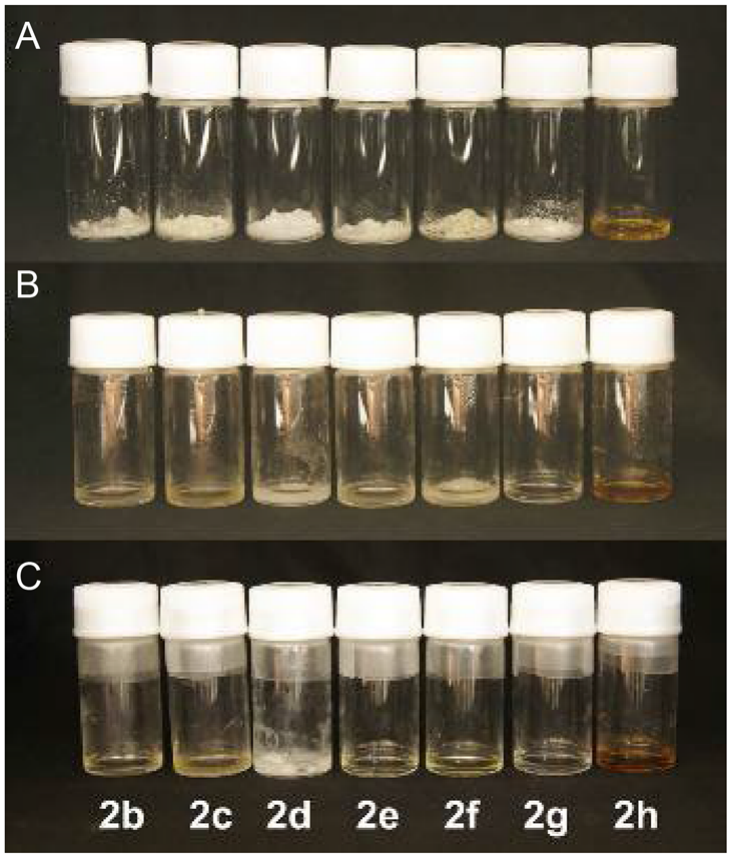
Receptors **2** and LiCl complex solutions A) before heating, B) immediately after heating, and C) after cooling for 1 week ([Receptor] = 3.0 M, LiCl 1 equiv., in MeCN).

Complex formations of receptors **2b** and **2g** with ion pairs were also studied. First, we checked the cooperative recognition of **2** for both Li^+^ and Cl^−^ from the changes in the ^1^H NMR spectra in MeCN‐*d*_3_ (**Figure** **3**). The urea NHs of receptor **2b** at around d 5.20 ppm showed a downfield shift to around 6.00 ppm upon the simultaneous addition of LiPF_6_ and TBACl, and the ether CH_2_ also showed a slight downfield shift of 0.04–0.06 ppm. These shifts are consistent with the changes in the respective functional groups that associate separately with the anions and the cations, suggesting that receptor **2b** is capable of binding these two ionic species simultaneously (Figure 3A). Such changes were also observed for **2g** with an aromatic substituent (Figure 3B), suggesting that both receptors are capable of ion pair recognition. Furthermore, the addition of one equivalent of LiCl to a solution of receptors **2b** and **2g**, rather than each of the independent ions, resulted in larger downfield shifts of NH groups, indicating ion‐pair recognition by receptors **2b** and **2g**. These differences are likely due to the influence of the counterion, suggesting true ion‐pair recognition in the presence of LiCl. Next, we examined the solubilization of LiCl with receptor **2** in organic solvents as observed with receptor **1**. **Figure** **4** represents the appearance of mixtures of receptor **2** and 1 equiv. of LiCl in MeCN ([**2**] = 3.0 M) before and after heating at 90 °C for 1 h and standing at r.t. for 1 week.^[^ ^21^ ^]^ A mixture of receptor **1a** and LiCl in MeCN remained solid before heating, and the mixture was dissolved after heating at 90 °C. Surprisingly, mixtures of receptors **2b**, **2c**, and **2g** and LiCl showed slightly dissolved with precipitations after mixing without heating and a mixture of **2h** and LiCl showed almost dissolved. Furthermore, for the mixture of receptors **2b**, **2c**, **2f**, **2g**, and **2h**, the LiCl complex solution was stable in the liquid state after heating, and no solidification was observed after more than 1 week of cooling. Although the **1a·**LiCl complex solution solidifies easily in about 1 day, the unsymmetrical structure of receptor **2** significantly suppressed the solidification of the solution with LiCl. These results suggest that the unsymmetrical structure of receptor **2** is quite important in maintaining the stable solution state.

### 2.4 Characterization of LiCl Complex Solution

The viscosity of the 3.0 M receptor**·**LiCl complex solution was measured to be 166.1 and 76.6 mPa**·**s for **1a·**LiCl and **2b·**LiCl, respectively (**Table** **4**). The viscosity of the 1.0 M solution was slightly lower (35.4 and 10.9 mPa**·**s for **1a·**LiCl and **2g·**LiCl, respectively). The reason for the high viscosity of 1.0 M **2b·**LiCl is the layer separation of the solution. Previous studies have revealed that even in complex solutions of **1a** and LiCl, the 0.5–2.0 M solution separates into two layers after standing for a certain time, forming a lower concentration solution (upper layer) and a highly viscous concentrated solution consisting of the receptor, LiCl, in solvent molecules in the lower layer.^[^ ^21^ ^]^ Although 1.0 M **2b·**LiCl complex in MeCN showed a layer separation similar to that of **1a·**LiCl, no layer separation formed for **2g·**LiCl, indicating that the **2g·**LiCl complex is highly solvated with the solvent molecules and is much advantageous for application in batteries and so on.

**Table 4:** Viscosity of **1a**·LiCl, **2b**·LiCl, and **2g**·LiCl complex solutions.

[Receptor**·**LiCl] [M]	*η* [mPa**·**s]			
	LiCl	**1a·**LiCl	**2b·**LiCl	**2g·**LiCl
0.025	5.51	–	–	–
0.25	–	5.65	6.71	5.98
1.0	–	35.4	112.4	10.9
3.0	–	166.1	76.6	232.7

**Figure 5:**
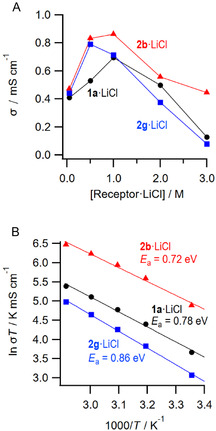
A) Ionic conductivity of **1a·**LiCl (•), **2b·**LiCl (▴), and **2g**·LiCl (▪) complex solution at each concentration (0.05–3.0 M) and B) Arrhenius plots of 3.0 M solution of receptor**·**LiCl in MeCN.

In addition, the ionic conductivity of the receptor·LiCl complex solutions was determined by AC impedance measurements (**Table** **5** and **Figure** **5**). At room temperature, *σ* = 0.409, 0.473, and 0.440 mS cm^−1^ for the 0.5 M **1a·**LiCl, **2b·**LiCl, and **2g·**LiCl solution in MeCN, respectively. These values are higher than that for 0.025 M LiCl solution in EC/DMC = 1: 1 (*σ* = 0.023 mS cm^−1^), confirming that the receptor·LiCl complex solutions enhance the ionic conductivity. These solutions showed similar viscosities. Therefore, the increase in the ionic conductivity is not due to an increase in the concentration of Li^+^ by the solubilization with the receptor rather than the lowering of the viscosities. The concentration dependence on the ionic conductivity study revealed that the ionic conductivities were increased by increasing the concentration and then gradually decreased to show the maxima at 0.5–1.0 M in all cases due to the increased concertation of Li^+^ and the increased viscosity of the mixture as shown in Figure 5A. The ionic conductivity of **2b·**LiCl and **2g·**LiCl showed higher value at 0.5 and 1.0 M, respectively, than those of **1a·**LiCl complex solutions. In addition, **2b·**LiCl at 3.0 M, which is the most concentrated solution in this study, showed the highest value than those of other receptor**·**LiCl solutions at the same concentration. From the Arrhenius plots for 3.0 M receptor**·**LiCl solutions (Figure 5b), the activation energies *E*_a_ were determined and the value for **2b·**LiCl showed a practically low value, suggesting the potential of receptor **2b** as a useful LIB additive.

**Table 5:** Ionic conductivity of receptor·LiCl composite solution at various concentrations.

[Receptor**·**LiCl] [M]	*σ* [mScm^−1^]			
	LiCl	**1a·**LiCl	**2b·**LiCl	**2g·**LiCl
0.025	0.023a)	–	–	–
0.05	–	0.409	0.473	0.440
0.50	–	0.529	0.832	0.790
1.0	–	0.695	0.862	0.715
2.0		0.498	0.558	0.376
3.0	–	0.128	0.444	0.078

a) Measured in EC/DMC = 1:1.

### 2.5 Solid–Liquid Extraction of LiCl

**Figure 6:**
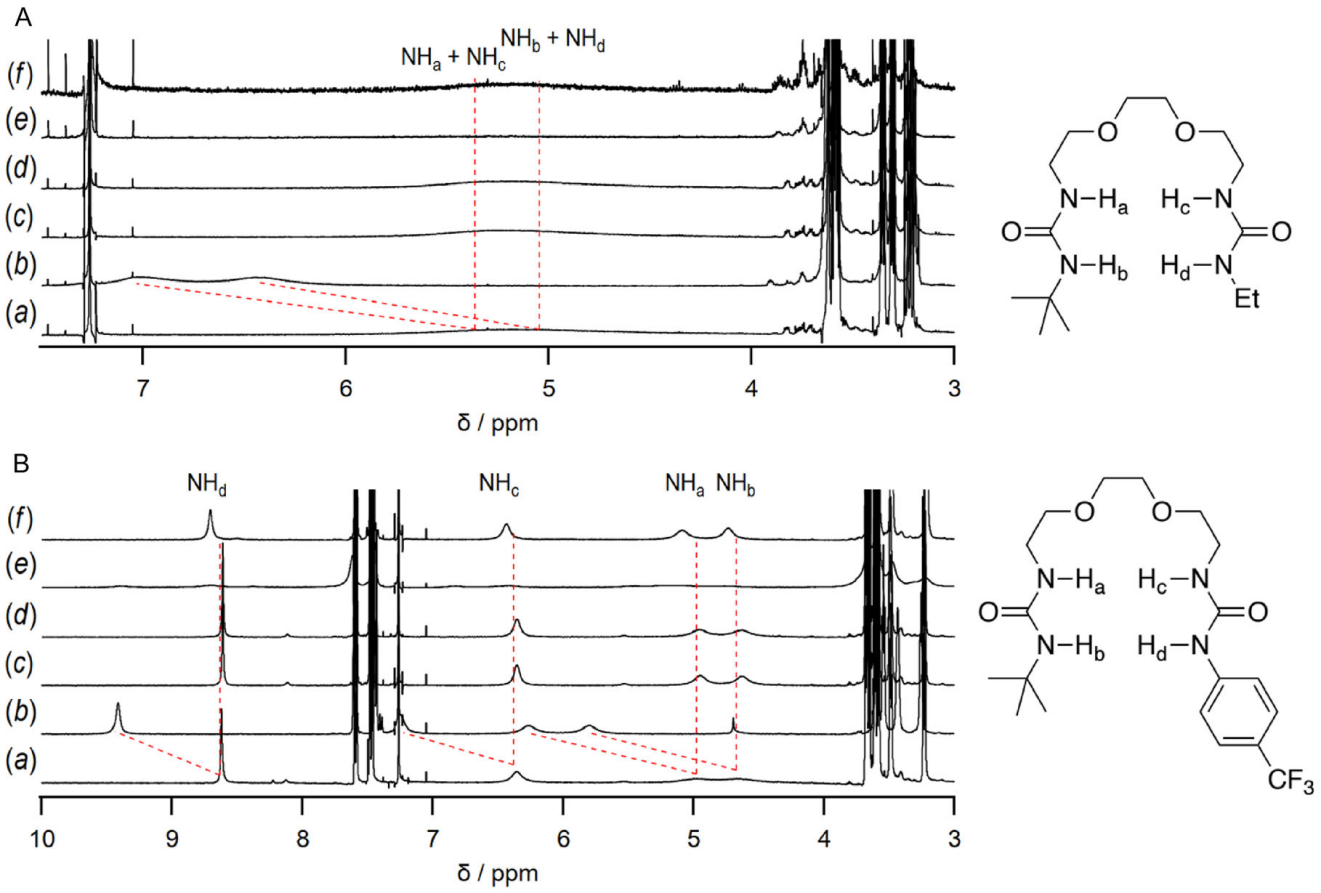
^1^H NMR spectra of solid–liquid extraction of A) **2b** and B) **2g** in CDCl_3_ in the presence of (a) in the absence and presence of (b) LiCl, (c) NaCl, (d) KCl, (e) MgCl_2_, and (f) CaCl_2_.

Finally, solid–liquid extraction studies of the receptors were also performed as an application of the LiCl solubilization. ^1^H NMR measurements of each receptor in the absence and presence of excess amounts of alkaline and alkali earth chloride salts, such as LiCl, NaCl, KCl, MgCl_2_, and CaCl_2_, in CDCl_3_ are shown in **Figure** **6**. NH protons of **2b** appeared broadly at d 5.17 ppm, and the peak showed a split and downfield shift to 6.43 and 7.01 ppm in the presence of LiCl. In addition, the methylene protons adjacent to the ether groups also showed a downfield shift from a sharp peak at 3.62 ppm to more complicated peaks at around 3.63 ppm, implying a similar association with both ions as in Figure 3A in MeCN‐*d*_3_. It should be mentioned that virtually no shifts were observed in the presence of other chloride salts. These results clearly indicate that **2b** is capable of selective solid–liquid extraction for LiCl. For **2g**, the four urea NHs showed clear downfield shifts from 4.66, 4.97, 6.32, and 8.62 ppm to 5.80, 6.26, 7.24, and 9.42 ppm, respectively, in the presence of LiCl, and a slight upfield shift of the ether methylene groups from 3.49 and 3.58 ppm to 3.54 and 3.44 ppm, respectively, indicating that **2g** was also selectively solid–liquid extractable for LiCl. The selectivity may be due to the characteristics of the receptor**·**salt complexes, as observed in Figure 4. The formed solution of MgCl_2_ with **2b** and **2g** is not stable, resulting in rapid solidification of the temporarily dissolved complex. In due course, a quantitative study will be conducted to evaluate the extraction efficiency and selectivity of the solid–liquid extraction of LiCl using unsymmetrical receptors **2** from artificial and natural salt mixtures.^[^ ^26^, ^33^, ^34^ ^]^

**Figure 7:**
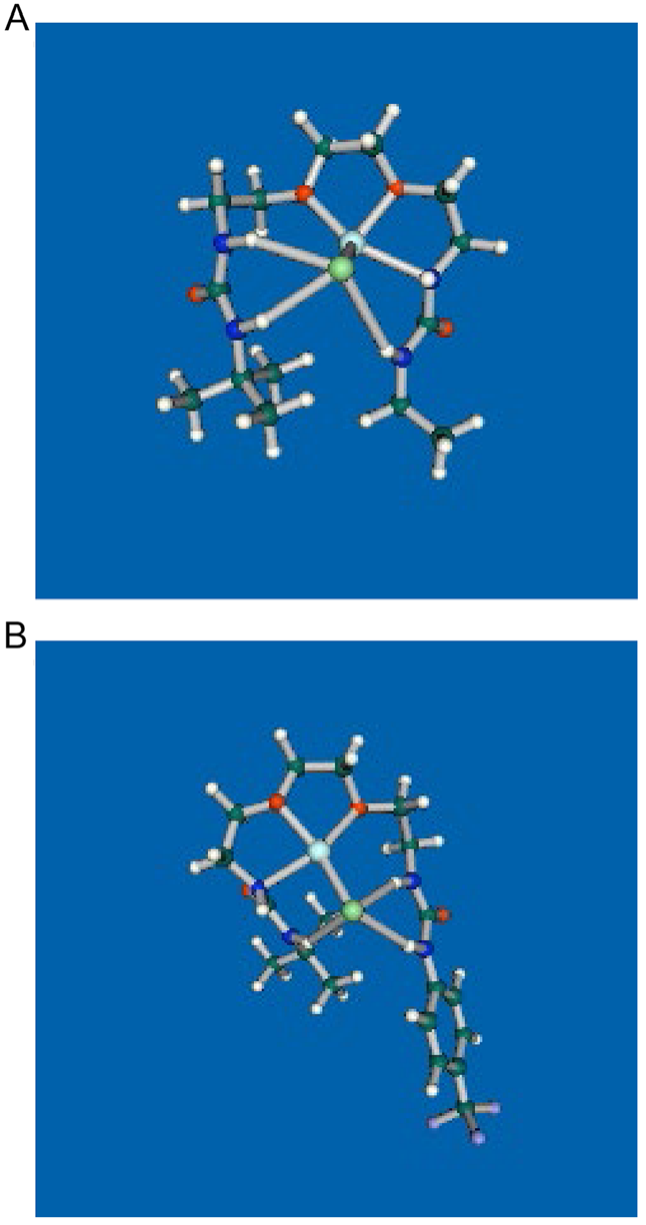
The optimized structures of A) **2b**·LiCl and B) **2g**·LiCl by DFT calculations (B3LYP‐D3/6‐31+G(d) level of theory) in CHCl_3_ (PCM).

The optimized structures of **2b·**LiCl and **2g·**LiCl were obtained by DFT calculations (B3LYP‐D3/6‐31+G(d) in CHCl_3_ (polarized continuum medium, PCM^[^ ^35^ ^]^) level of theory) as depicted in **Figure** **7**. In both cases, Li^+^ is coordinated by two ether oxygens and one sp^3^ nitrogen atom of the urea group bearing ethyl and *t*‐butyl groups for **2b** and **2g**, respectively. Four NH groups form hydrogen bonds to one Cl^−^. For **2b·**LiCl, interatomic distances of NHs and Cl^−^ are almost similar (Figure 7A, NH_a_‐Cl: 2.51, NH_b_‐Cl: 2.50, and NH_c_‐Cl: 2.53) except for the sp^3^ nitrogen (NH_d_‐Cl: 2.77 Å), whereas for **2g·**LiCl, the urea conjugated with the terminal substituent forms a stronger hydrogen bond (Figure 7B, NH_a_‐Cl: 2.83, NH_b_‐Cl: 2.66, NH_c_‐Cl: 2.48, and NH_d_‐Cl: 2.29 Å). This is due to the formation of strong hydrogen bonds by the more acidic NH group of the urea bearing the phenyl group with the electron‐withdrawing substituent.

## 3 Conclusion

In conclusion, we have presented a series of unsymmetrical receptors **2** that can effectively solubilize LiCl at higher concentrations than those of the symmetrical analogue **1**. The receptors were easily prepared in two steps from the commercially available starting materials. The receptors, especially **2g** and **2h**, exhibited suitable properties such as high solubility in organic solvents due to the bulky aliphatic substituents and strong association ability and selectivity for Cl^−^ due to another aromatic substituent. In addition, receptor **2** can solubilize LiCl up to 3.0 M, and the solution has sufficient ionic conductivity, low viscosity, and less solidifying property resulting in high potential for applications such as an electrolyte for LIB. Furthermore, the ion‐pair recognition of **2** was highly LiCl selective; then, the solid–liquid extraction of LiCl from a salt mixture was also possible. This selectivity was attributed to the stability of the complex in solution. This remarkable LiCl selectivity of receptors **2** can also be applied to LiCl recovery from natural resources. These results clearly indicate that the symmetry‐breaking strategy is significantly useful for maintaining the solution and liquid states of a complex in materials chemistry.

## 4 Experimental Section

All reagents used were of analytical grade. All salts were purchased as anhydrous and were handled under a nitrogen atmosphere. NMR spectra were measured on a JEOL ECZ‐500 R (500 MHz) spectrometer. Electrospray ionization mass spectrometry (ESI‐MS) were measured on an Agilent 6200 series TOF. Viscosity was measured with a Tokai Sangyo Viscometer TV‐22. AC impedance measurements were performed by a Solatron Analytical 1260.

4.1

4.1.1

##### Synthesis of 1‐(2‐(3‐Tert‐Butylureidoethoxy))‐2‐(2‐Amionoethoxy)ethane (3)

Into a solution of diamine (11.11 g, 75.0 mmol, 4.84 equiv.) in THF (130 mL), *tert*‐butyl isocyanate (1.77 mL, 15.5 mmol) in THF (65 mL) was dropwised at 0 °C under argon atmosphere. The resulting mixture was stirred at 0 °C for 2 h. After evaporation of the mixture under reduced pressure, the residue was dissolved in CHCl_3_ (45 mL), and the solution was extracted with saturated aqueous ammonium chloride (45 mL × 3). The combined aqueous phase was made basic (pH 10) with sodium hydrogen carbonate, and the resulting alkaline solution was extracted with chloroform (135 mL × 3). The combined organic phase was dried over anhydrous sodium sulfate and evaporated under reduced pressure to give the product as opalic viscous oil. Yield 2.75 g, 74%. ^1^H NMR (500 MHz, CDCl_3_) *δ* 5.27 (s, 1H), 5.20 (s, 1H), 3.63 (s, 4H), 3.56 (t, 2H, *J* = 4.5 Hz), 3.55 (t, 2H, *J* = 4.5 Hz), 3.34 (q, 2 H, *J* = 5.0 Hz), 2.91 (t, 2 H, *J* = 5.0 Hz), 2.17 (s, 2H), 1.32 (s, 9H). ^13^C NMR (126 MHz, CDCl_3_) *δ* 158.0, 72.3, 70.7, 70.0, 69.8, 50.1, 41.4, 39.8, 29.5. HRMS (ESI^+^): Calcd for C_11_H_26_N_3_O_3_ [M + H]^+^, 248.1969. Found 248.1976.

##### Typical Procedure for Preparation of Receptors 2

Into a solution of **3** (1.0 g, 4.0 mmol) in THF (25 mL), appropriate isocyanate (5.0 mmol) was added dropwise via syringe under an argon atmosphere, and the mixture was refluxed for 5 h. The mixture was evaporated under reduced pressure, and the residue was washed with ethyl acetate (**2a**), recrystallized from ethyl acetate–hexane (**2b**–**d**) or methanol (**2h**), and chromatographed on silica gel with 5% MeOH/dichloromethane (**2e**–**g**).

##### Saturated Concentration of Receptors

A saturated solution of the receptor in CDCl_3_ and MeCN‐*d*_3_ was prepared. An appropriate amount of the solution was added into an NMR tube via a microsyringe, and the solution was evaporated under reduced pressure. Into the NMR tube, 600 μL of naphthalene (2.0 mM) solution in CDCl_3_ was added and the NMR was measured. From the integration of the receptor and naphthalene, the saturation concentration was determined.

##### NMR Titrations of Receptor with Cation and Anion

A solution of receptor was prepared ([receptor] = 10 mM in MeCN‐*d*_3_) and filled into an NNR tube; then, the ^1^H NMR was measured. An aliquot of stock solution of guest salts (TBACl, TBAAcO, LiPF_6_, and NaPF_6_) in MeCN‐*d*_3_ was added to the NMR tube, followed by ^1^H NMR of the mixture was measured. The process was repeated to obtain the titration data. The association constants were calculated from the data by BindFit (see http://supramolecular.org).^[^ ^36^ ^]^ The titrations were performed in at least triplicate to ensure accuracy of the results.

##### UV–Vis Titrations of Receptor with Cation and Anion

A solution of the receptor was prepared ([receptor] = 2.0 × 10^−5^ M) in MeCN and placed in a UV cuvette. Then, the UV spectrum of the solution was measured. An aliquot of the stock solution of guest salts (TBACl and TBAAcO) in MeCN was added to the cuvette. Then, the UV spectrum of the mixture was measured. This process was repeated to obtain the titration data. The association constants were calculated from the data by BindFit (see http://supramolecular.org).^[^ ^36^ ^]^ The titrations were performed in at least triplicate to ensure accuracy.

##### Solid–Liquid Extraction of Inorganic Salts with Receptors in CDCl_3_

A solution of receptor was prepared ([receptor] = 10 mM in CDCl_3_). Into an NMR tube, 500 μL of the solution was added, and the NMR was measured. Finely powdered solid salt (100 eq.) was added into the NMR tube, the mixture was stirred vigorously at room temperature, and the NMR was measured again.

##### Measurement of Viscosity

The viscosity of acetonitrile solutions of **1a·**LiCl, **2b·**LiCl, and **2g·**LiCl (0.25, 1.0, and 3.0 M) was measured with a viscometer (Viscometer TV‐22, Toki Sangyo Co., Ltd., Japan). The viscosity of 0.025 M LiCl solutions was also measured. A sample (1 mL) was loaded, and the viscosity was obtained at a rotational speed of 50 rpm under 25 °C after standing for 2 min to stabilize the temperature. The results are summarized in Table 4.

##### Measurement of Ionic Conductivity

A solution (3 M) of receptor and LiCl in MeCN was placed in a two‐terminal cell and the impedance was measured. The impedance of the solution was calculated from the resistance determined from the Cole–Cole plot and the cell constant (0.2826 cm^−1^).

##### DFT Calculations

The optimized structures of **2b·**LiCl and **2g·**LiCl were performed by Gaussian 16, Revision A.03,^[^ ^37^ ^]^ at the B3LYP‐D3/6‐31+G(d) level of theory in chloroform (PCM). The energetically lowest structures are shown in Figure 7, and the Cartesian coordinates of the structure are shown in Table S2 and S3, Supporting Information, respectively.

## Conflict of Interest

The authors declare no conflict of interest.

## Supporting information

Supplementary Material

## Data Availability

The data that support the findings of this study are available in the Supporting Information of this article.
