# Supplementary material for: Low‐Viscosity Concentrated Lithium Chloride Solution with Unsymmetrical Ditopic Receptors in Organic Solvents
Source: Chemphyschem. 2025 Oct 26;26(24):e202500601. doi: 10.1002/cphc.202500601 (PMC12710136; doi:10.1002/cphc.202500601)
Supplement: Supplementary file 1 — Supplementary Material [file CPHC-26-e202500601-s001.pdf]

Supporting Information

Contents

General ········ S2
Synthesis of receptors ········ S2
Saturation concentration of receptors ········ S22
NMR titrations ········ S22
UV-vis titrations ········ S24
Solid-liquid and liquid-liquid extraction of salts by NMR ········ S26
Measurement of viscosity ········ S26
Measurement of ionic conductivity ········ S26
DFT calculations ········ S26
References ········ S29

General

All reagents used were of analytical grade. All salts were purchased as anhydrous and were handled under a nitrogen atmosphere. NMR spectra were measured on a JEOL ECZ-500R (500 MHz) spectrometer. ESI-MS were measured on an Agilent 6200 series TOF. Viscosity was measured with a Tokai Sangyo Viscometer TV-22. AC impedance measurements were performed by a Solatron Analytical 1260. Receptors **1a**, **1d**, and **1f** were prepared according to the literature. [1]

Synthesis of receptors

1,2-Bis(2- (3-ethylureido)ethoxy)ethane (1b)

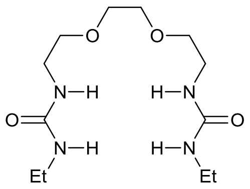

A mixture of 1,2-bis(2-aminoethoxy)ethane 1.00 g (6.77 mmol) and ethyl isocyanate 1.16 mL (14.9 mmol, 2.2 eq) in dry THF (5 mL) was refluxed under an argon atmosphere for 3 h. The mixture was evaporated under reduced pressure and the residue was recrystallized from ethyl acetate giving colorless solids as the product (1.94 g, 98%). Mp. 137.6-140.1 °C. ^{1}H NMR (500 MHz, $CDCl_3$) δ 5.45 (s, 4H), 3.61 (d, 2H, *J* = 5.7 Hz), 3.62 (s, 4H), 3.58 (t, 4H, *J* = 5.1 Hz), 3.35 (t, 4H, *J* = 5.1 Hz), 3.21 (q, 4H, *J* = 7.2 Hz), 1.12 (t, 6H, *J* = 7.2 Hz). ^{13}C NMR (126 MHz, $CDCl_3$) δ 159.0, 70.7, 70.3, 40.3, 35.0, 15.5. HRMS (ESI^+): Calcd for $C_{12}H_{26}N_4O_4Na$ $[M+Na]^+$, 313.1846. Found 313.1829.

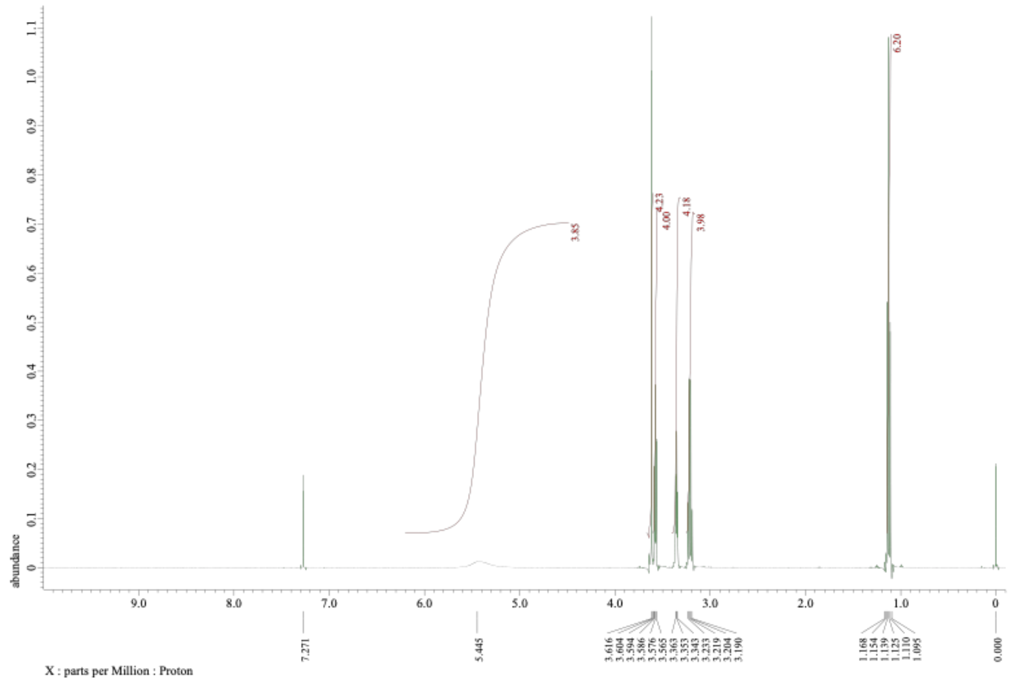

Fig. S1. ^{1}H NMR spectrum (500 MHz) of receptor **1b** in $CDCl_3$.

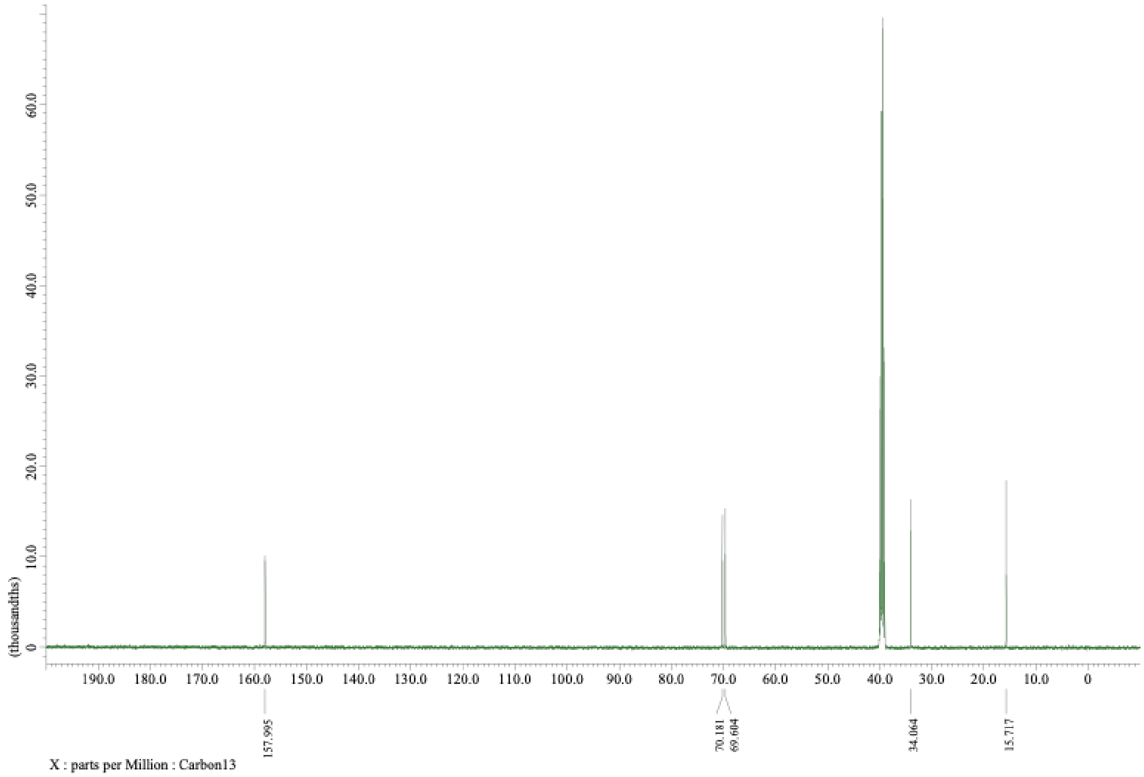

Fig. S2. ^{13}C NMR spectrum (126 MHz) of receptor **1b** in $CDCl_3$.

1,2-Bis(2-(3-*iso*-propylureido)ethoxy)ethane (1c)

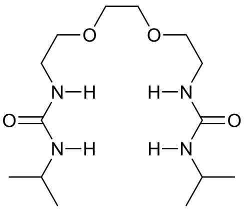

A mixture of 1,2-bis(2-aminoethoxy)ethane 1.00 g (6.75 mmol) and *i*-propyl isocyanate 1. 57 mL (16.0 mmol, 2.2 eq) in dry THF (5 mL) was refluxed under an argon atmosphere for 3 h. The mixture was evaporated under reduced pressure and the residue was recrystallized from ethyl acetate giving colorless solids as the product. (1.88 g, 86%). Mp. 145.0-148.1 °C. ^{1}H NMR (500 MHz, $CDCl_3$) δ 5.54 (s, 2H), 5.05 (s, 2H), 3.89 (sept, 2H, *J* = 6.4 Hz), 3.62 (s, 4H), 3.58 (t, 4H, *J* = 5.0 Hz), 3.34 (t, 4H, *J* = 5.00 Hz), 1.14 (d, 12H, *J* = 6.4 Hz). ^{13}C NMR (126 MHz, DMSO-d_6) δ 157.4, 70.2, 69.6, 40.9, 39.1, 23.3. HRMS (ESI^+): Calcd for $C_{14}H_{30}N_4O_4Na$ $[M+Na]^+$, 341.2159. Found 3491.2151.

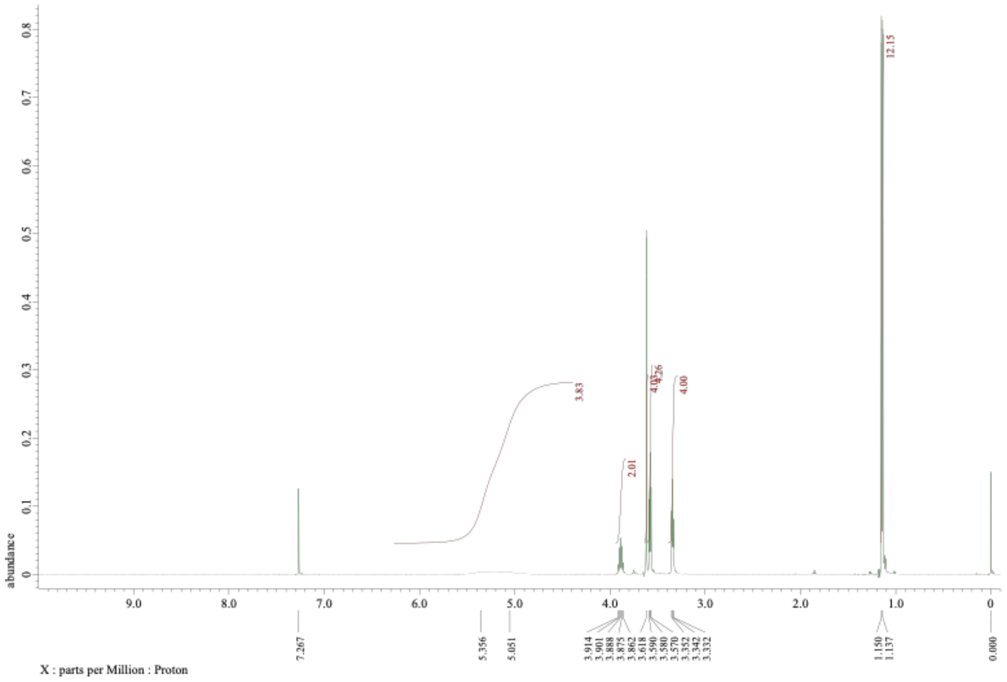

Fig. S3. ^{1}H NMR spectrum (500 MHz) of receptor **1c** in $CDCl_3$.

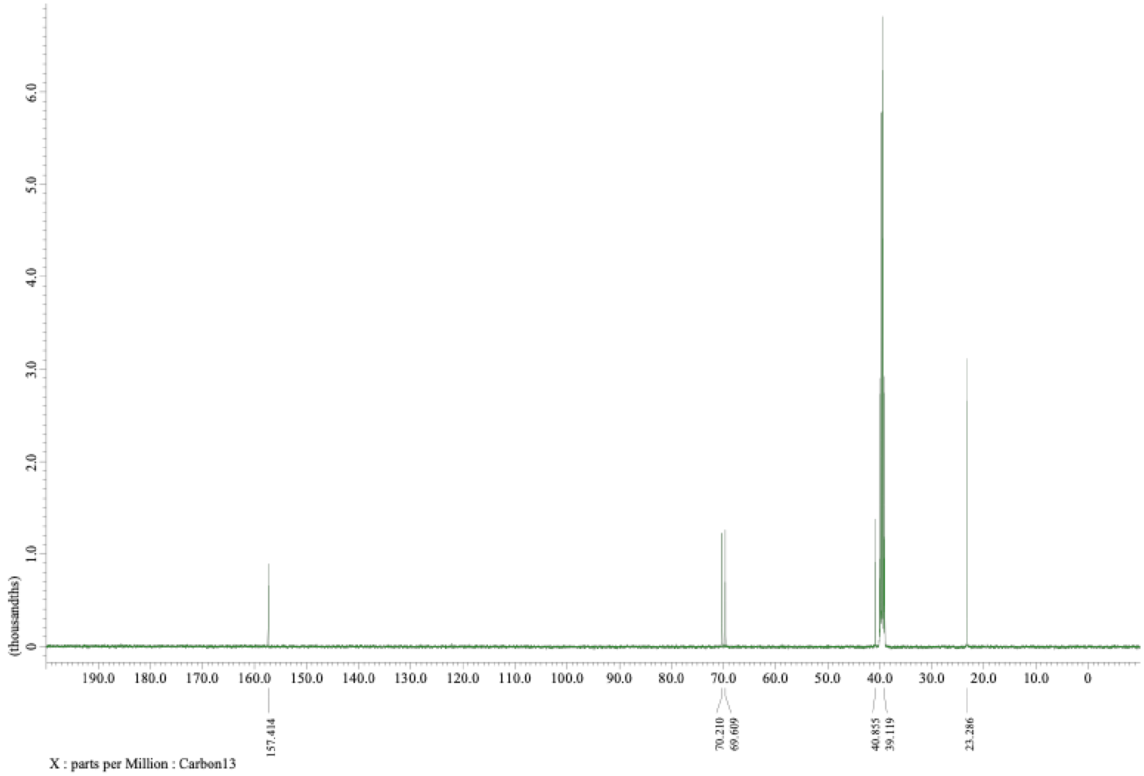

Fig. S4. ^{13}C NMR spectrum (126 MHz) of receptor **1c** in $CDCl_3$.

1,2-Bis(2-(3-dodecylureido)ethoxy)ethane (1e)

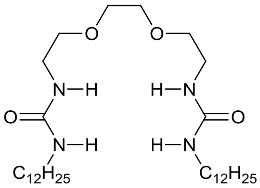

A mixture of 1,2-bis(2-aminoethoxy)ethane (337 mg, 2.27 mmol) and dodecyl isocyanate (1.17 mg, 2.2 eq) in dry THF (30 mL) was refluxed under an argon atmosphere for 6 h. The mixture was evaporated under reduced pressure. The residue was recrystallized from AcOEt to give colorless fiber-like solids as the product. (1.16 g, 89%). M.p. 135.5-136.0 °C. ^{1}H NMR (500 MHz, $CDCl_3$) δ 5.27 (br s, 2H), 5.16 (br s, 2H), 3.62 (s, 4H), 3.58 (t, 4H, *J* = 4.9 Hz), 3.35 (t, 4H, *J* = 4.9 Hz), 3.16 (t, 4H, *J* = 7.2 Hz), 1.48 (quint, 4H, *J* = 7.0 Hz), 1.32–1.23 (m, 36H), 0.88 (t, 6H, *J* = 7.0 Hz). ^{13}C NMR (126 MHz, $CDCl_3$) δ 159.1, 70.8, 70.4, 40.6, 40.6, 32.1, 30.5, 29.8, 29.8, 29.6, 29.5, 27.1, 22.8, 14.3. HRMS (ESI$^+$): Calcd for $C_{32}H_{66}N_4O_4Na$ $[M+Na]^+$, 593.4976. Found 593.4974.

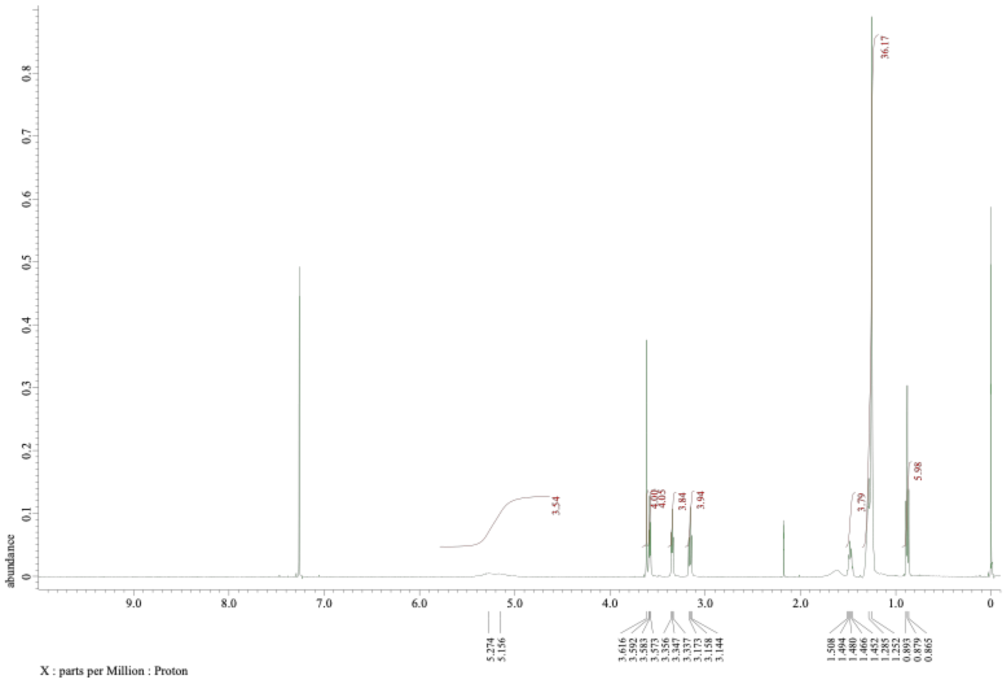

Fig. S5. ^{1}H NMR spectrum (500 MHz) of receptor **1e** in $CDCl_3$.

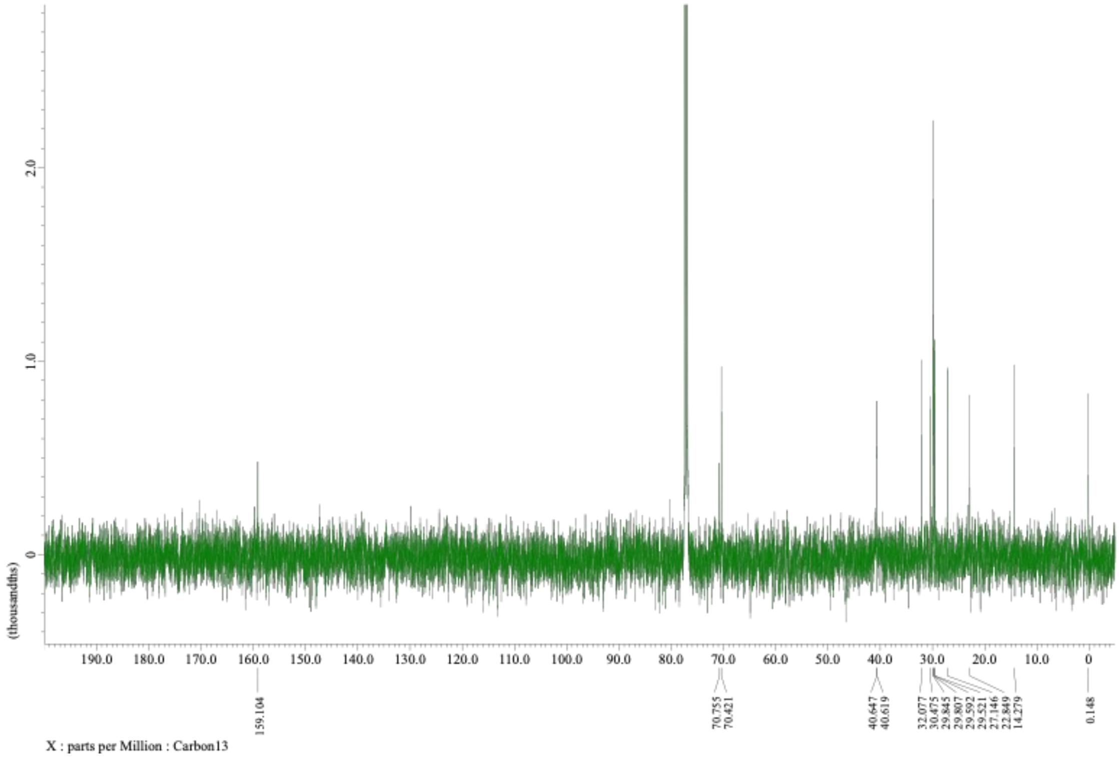

Fig. S6. ^{13}C NMR spectrum (126 MHz) of receptor **1c** in $CDCl_3$.

1,2-Bis(2-(3-(4-trifluoromethylphenyl)ureido)ethoxy)ethane (1g)

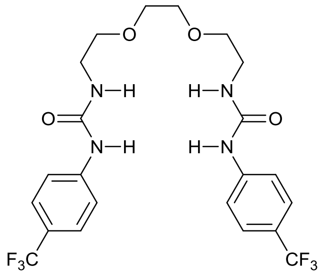

A mixture of 1,2-bis(2-aminoethoxy)ethane (1.01 g, 6.78 mmol) and 4-(trifluoromethyl)phenyl isocyanate (2.1 mL, 2.2 eq) in dry THF (5 mL) was refluxed under an argon atmosphere overnight. The mixture was evaporated under reduced pressure. The residue was recrystallized from AcOEt to give colorless fiber-like solids as the product (1.97 g, 56%). 148.5–150.5 °C. ^{1}H NMR (500 MHz, DMSO-d_6) δ 8.98 (s, 2H), 7.56 (d, 4H, J = 9.1 Hz), 7.54 (d, 4H, J = 9.1 Hz), 6.33 (t, 2H, J = 5.5 Hz), 3.55 (s, 4H), 3.46 (t, 4H, J = 5.5 Hz), 3.26 (q, 4H, J = 5.5 Hz). ^{13}C NMR (126 MHz, DMSO-d_6) δ 154.8, 144.2, 126.0 (q, $^{3}J_{CF}$ = 4.0 Hz), 124.7 (q, $^{1}J_{CF}$ = 272 Hz), 121.0 (q, $^{2}J_{CF}$ = 32 Hz), 117.2, 66.7, 69.6, 39.0. HRMS (ESI$^+$): Calcd for $C_{22}H_{24}N_4O_4F_6Na$ [M+Na]$^+$, 545.1594. Found 545.1593.

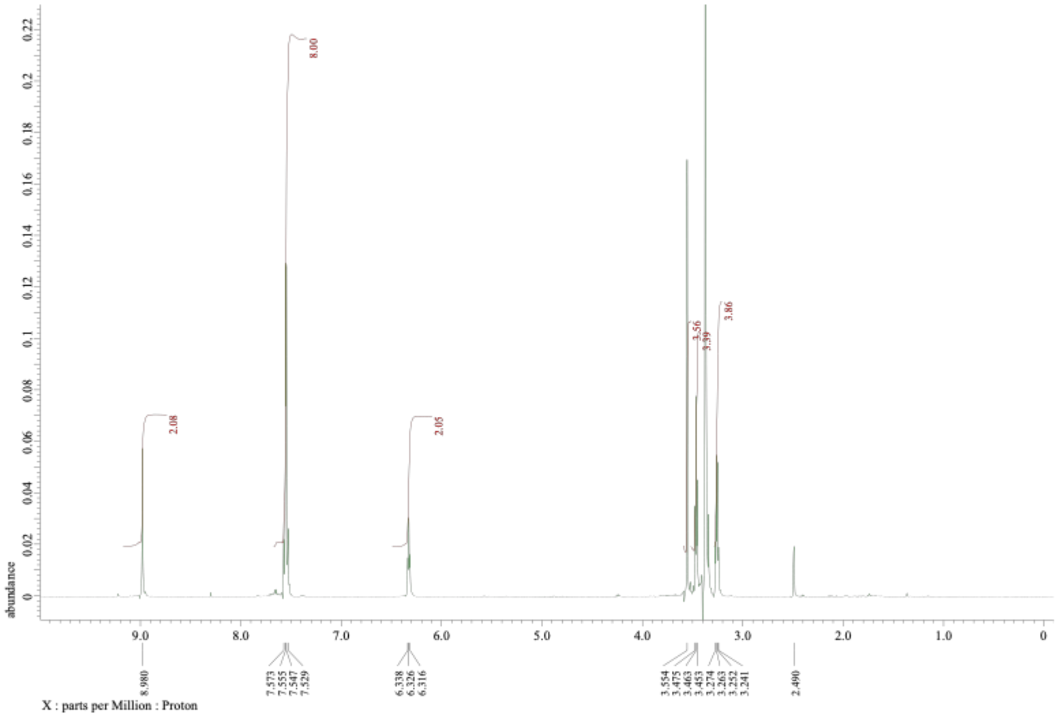

Fig. S7. ^{1}H NMR spectrum (500 MHz) of receptor **1g** in DMSO-d_6.

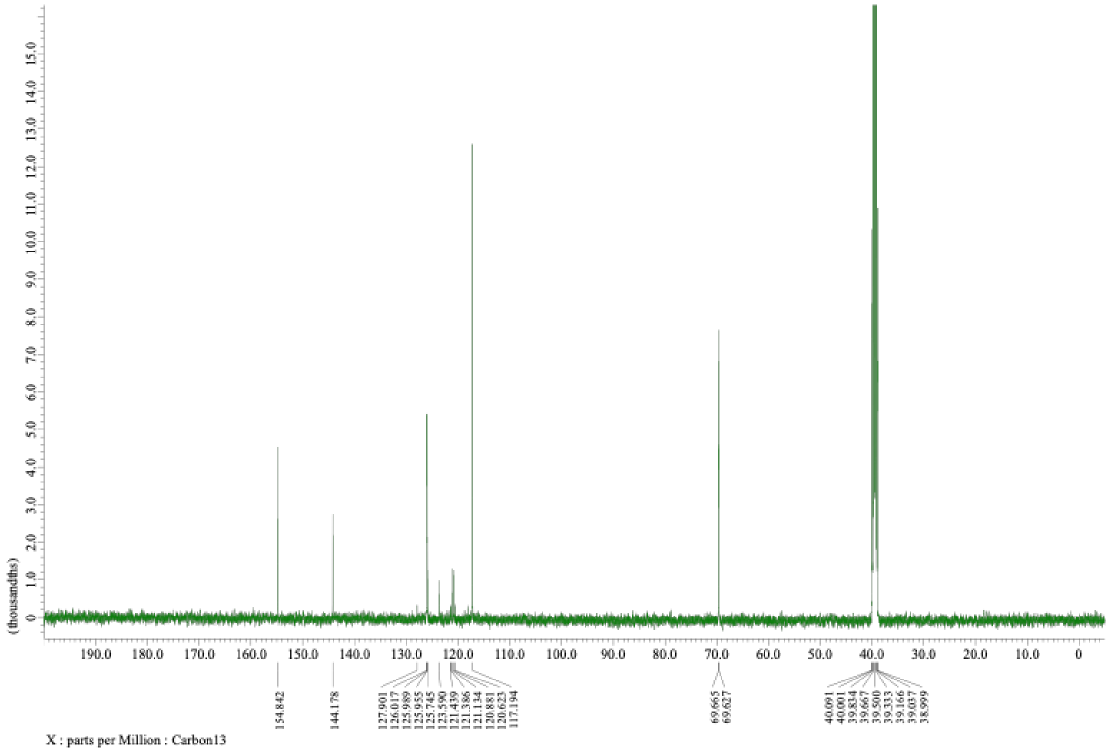

Fig. S8. ^{13}C NMR spectrum (126 MHz) of receptor **1g** in DMSO-d_6.

1,2-Bis(2-(3-(4-nitrophenyl)ureido)ethoxy)ethane (1h)

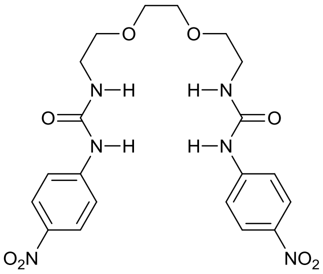

A mixture of 1,2-bis(2-aminoethoxy)ethane (1.01 g, 6.78 mmol) and 4-nitrophenyl isocyanate (2.43 g, 2.2 eq) in dry THF (4 mL) was refluxed under an argon atmosphere for 3 h. The mixture was evaporated under reduced pressure. The residue was recrystallized from AcOEt to give colorless fiber-like solids as the product (2.64 g, 82%). M.p. 170.0–171.0 °C. ^{1}H NMR (500 MHz, DMSO-d_6) δ 9.34 (s, 2H), 8.10 (dd, 4H, J_1 = 9.0, J_2 = 1.7 Hz), 7.59 (dd, 4H, J_1 = 9.0, J_2 = 1.7 Hz), 6.47 (t, 2H, J = 5.0 Hz), 3.55 (s, 4H), 3.47 (t, 4H, J = 5.0 Hz), 3.27 (q, 4H, J = 5.0 Hz). ^{13}C NMR (126 MHz, DMSO-d_6) δ 154.4, 147.1, 140.4, 125.2, 116.8, 69.6, 69.5, 39.1. HRMS (ESI$^+$): Calcd for $C_{20}H_{24}N_6O_8Na$ [M+Na]$^+$, 4991548. Found 499.1536.

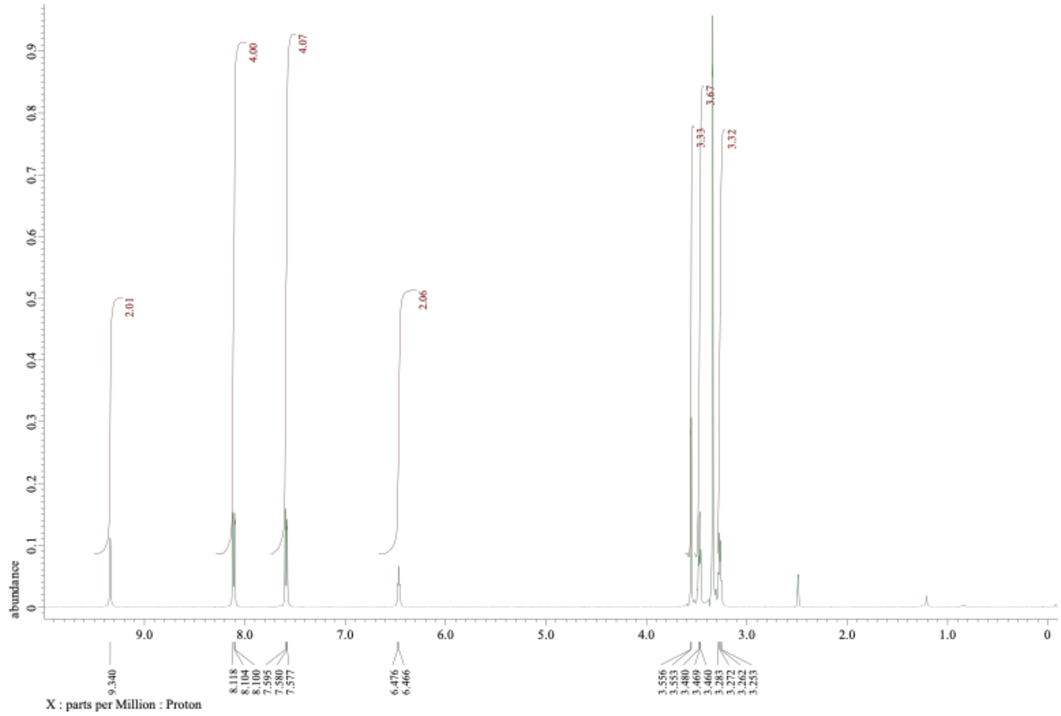

Fig. S9. ^{1}H NMR spectrum (500 MHz) of receptor **1h** in DMSO-d_6.

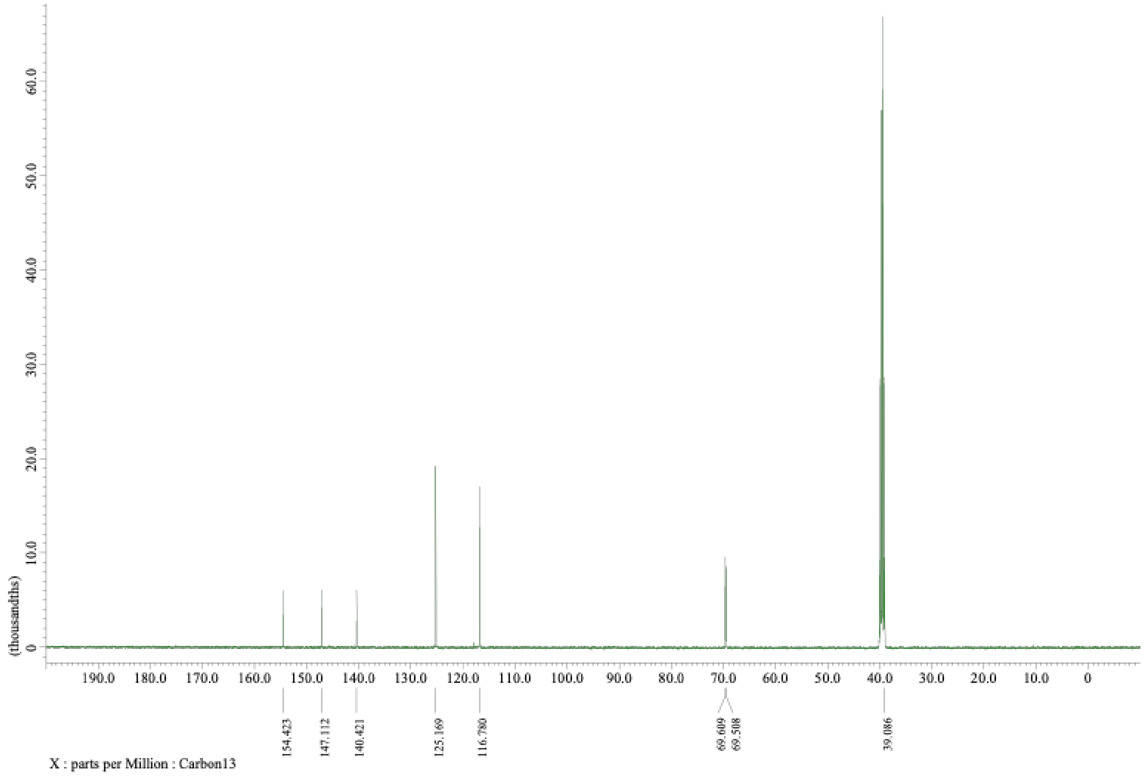

Fig. S10. ^{13}C NMR spectrum (126 MHz) of receptor **1f** in DMSO-d_6.

1-(2-(3-*tert*-Butylureidoethoxy))-2-(2-amionoethoxy)ethane (3)

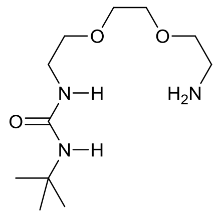

Into a solution of diamine (11.11 g, 75.0 mmol, 4.84 equiv) in THF (130 mL), *tert*-butyl isocyanate (1.77 mL, 15.5 mmol) in THF (65 mL) was dropwised at 0 °C under an argon atmosphere. The resulting mixture was stirred at 0 °C for 2 h. After evaoration of the mixture under reduced pressure, the residue was dissolveed in $CHCl_3$ (45 mL) and the solution was extracted with saturated aqueous ammonium chloride (45 mL × 3). The combined aqueous phase was basicify (pH 10) with sodium hydrogen carbonate and the resulting alkaline solution was extracted with chloroform (135 mL × 3). The combined organic phase was dried over anhydrous sodium sulfate and evaporated under reduced pressure to give the product as opalic viscous oil. Yield 2.75 g, 74%. ^{1}H NMR (500 MHz, $CDCl_3$) δ 5.27 (s, 1H), 5.20 (s, 1H), 3.63 (s, 4H), 3.56 (t, 2H, *J* = 4.5 Hz), 3.55 (t, 2H, *J* = 4.5 Hz), 3.34 (q, 2H, *J* = 5.0 Hz), 2.91 (t, 2H, *J* = 5.0 Hz), 2.17 (s, 2H), 1.32 (s, 9H). ^{13}C NMR (126 MHz, $CDCl_3$) δ 158.0, 72.3, 70.7, 70.0, 69.8, 50.1, 41.4, 39.8, 29.5. HRMS (ESI$^+$): Calcd for $C_{11}H_{26}N_3O_3$ $[M+H]^+$, 248.1969. Found 248.1976.

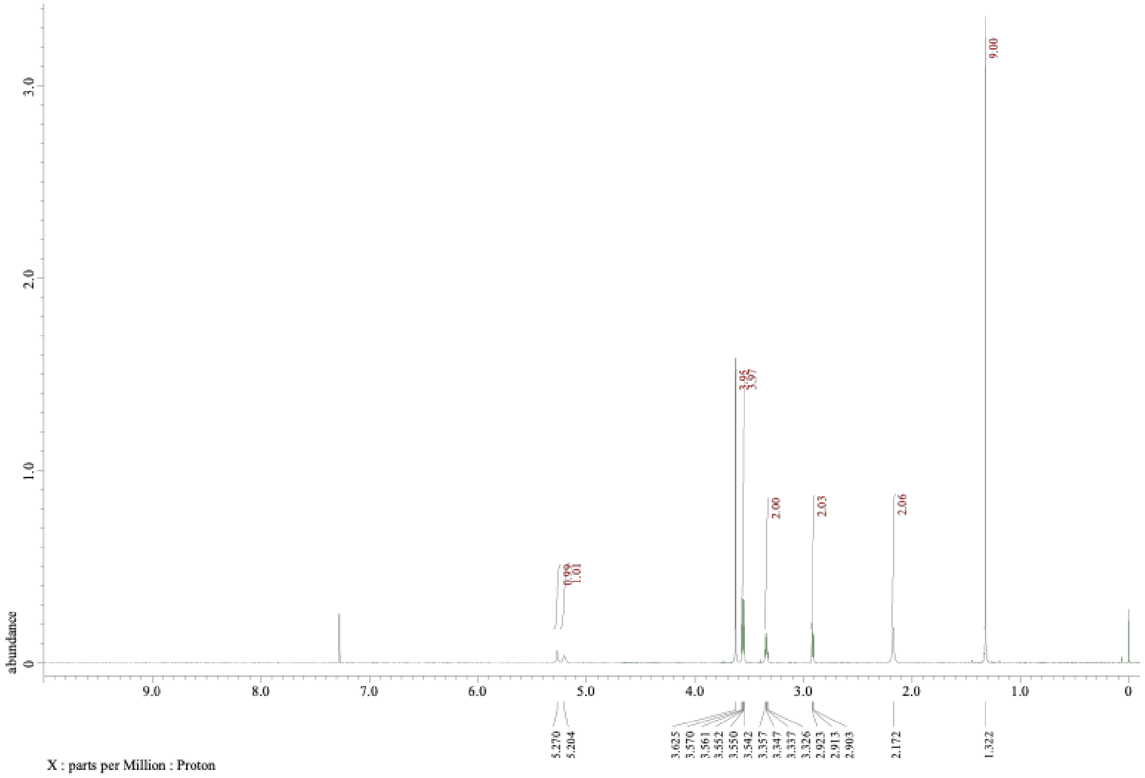

Fig. S11. ^{1}H NMR spectrum (500 MHz) of receptor **3** in $CDCl_3$.

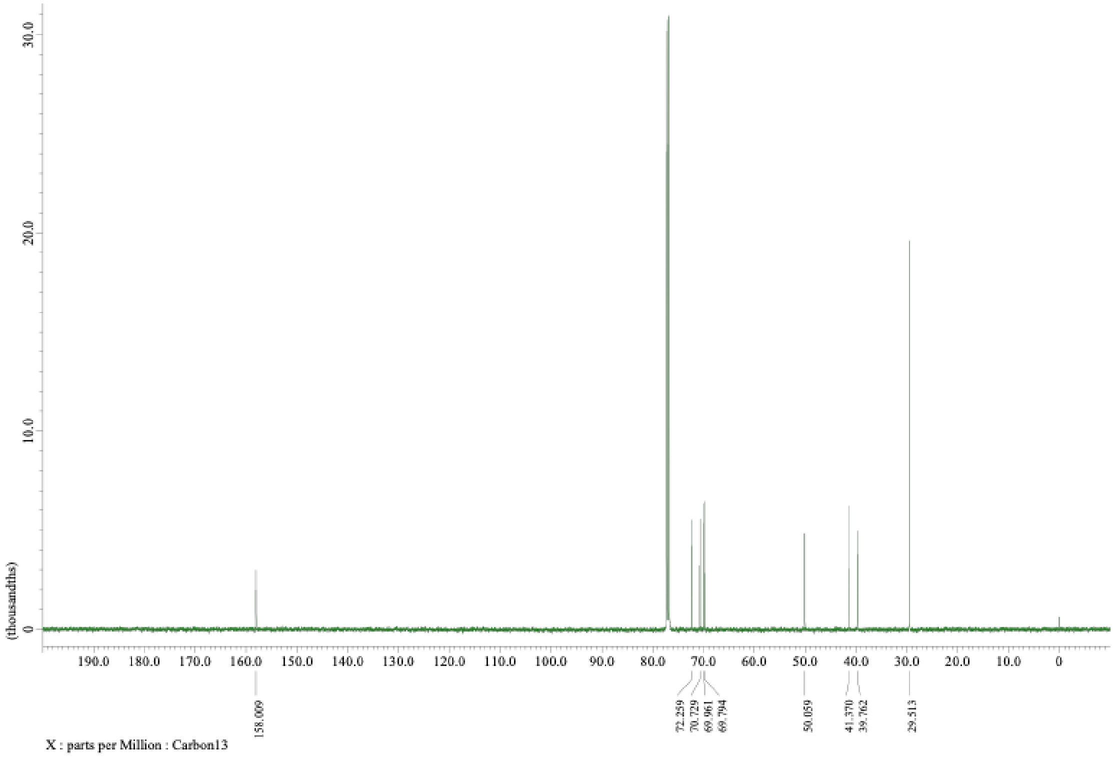

Fig. S12. ^{13}C NMR spectrum (126 MHz) of receptor **3** in $CDCl_3$.

Typical procedure for preparation of receptors 2

Into a solution of **3** (1.0 g, 4.0 mmol) in THF (25 mL), appropriate isocyanate (5.0 mmol) was added dropwise via syringe under an argon atmosphere, the mixture was refluxed for 5 h. The mixture was evaporated under reduced pressure and the residue was washed with ethyl acetate (**2a**), recrystallized from ethyl acetate–hexane (**2b-d**) or methanol (**2h**), and chromatographed on silica gel with 5% MeOH/dichloromethane (**2e-g**).

1-(2-(3-*tert*-Butylureido)ethoxy)-2-(2-(3-ethylureido)ethoxy)ethane (2b)

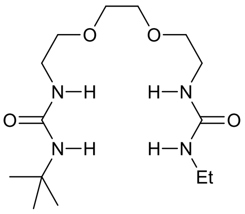

Yield 89%. M. p. 77.0–79.0 °C. ^{1}H NMR (500 MHz, $CDCl_3$) δ 5.62 (bs, 1H), 5.48 (bs, 1H), 5.41 (bs, 1H), 5.26 (bs, 1H), 3.61 (s, 4H), 3.57 (t, 2H, *J* = 5.1 Hz), 3.56 (t, 2H, *J* = 5.1 Hz), 3.35 (t, 2H, *J* = 5.0 Hz), 3.30 (t, 2H, *J* = 5.0 Hz), 3.21 (q, 2H, *J* = 7.3 Hz), 1.33 (s, 9H), 1.12 (t, 3H, *J* = 7.3 Hz). ^{13}C NMR (126 MHz, $CDCl_3$) δ 159.0, 158.3, 70.7, 70.7, 70.3, 70.2, 50.1, 40.3, 40.1, 35.0, 29.5, 15.5. HRMS (ESI$^+$): Calcd for $C_{14}H_{31}N_4O_4$ [M+H]$^+$, 319.2347. Found 319.2345.

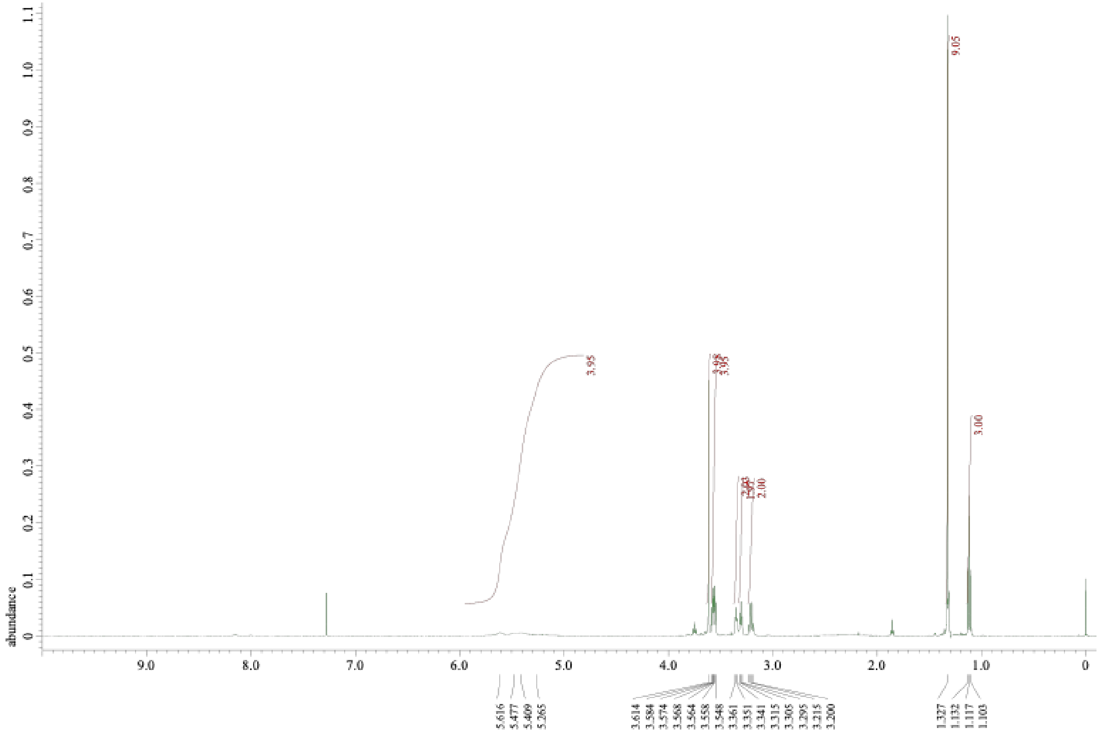

Fig. S13. ^{1}H NMR spectrum (500 MHz) of receptor **2b** in $CDCl_3$.

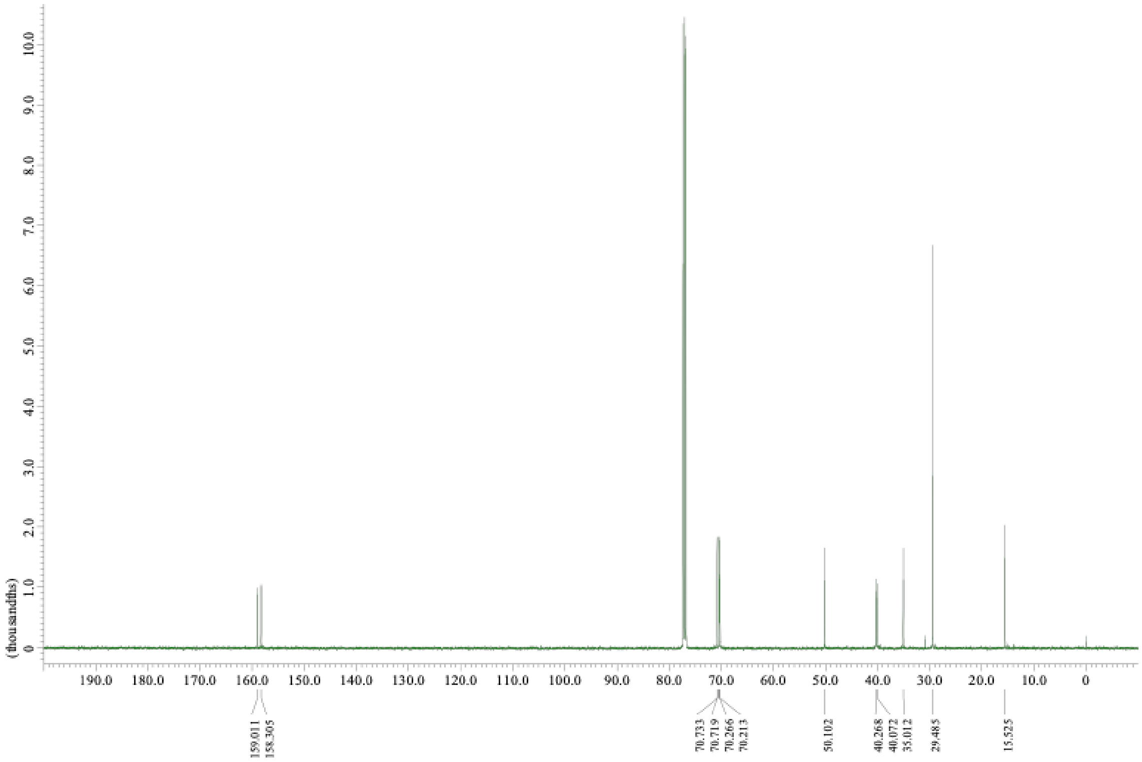

Fig. S14. ^{13}C NMR spectrum (126 MHz) of receptor **2b** in $CDCl_3$.

1-(2-(3-*tert*-Butylureido)ethoxy)-2-(2-(3-*iso*-propylureido)ethoxy)ethane (2c)

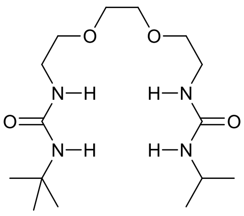

Yield 70%. M. p. 132.0–133.0 °C. ^{1}H NMR (500 MHz, $CDCl_3$) δ 5.40 (s, 1H), 5.32 (s, 1H), 5.10 (s, 1H), 4.97 (d, 1H, J = 6.5 Hz), 3.89 (qd, 1H, J = 6.5 Hz), 3.62 (s, 4H), 3.59 (t, 2H, J = 5.0 Hz), 3.57(t, 2H, J = 5.0 Hz), 3.34 (q, 2H, J = 5.0 Hz), 3.30 (q, 2H, J = 5.0 Hz), 1.34 (s, 9H), 1.14 (d, 6H, J = 6.5 Hz). ^{13}C NMR (126 MHz, $CDCl_3$) δ 158.3, 158.2, 70.7, 70.3, 70.2, 50.1, 41.9, 40.3, 40.1, 29.5, 23.4. HRMS (ESI^{+}): Calcd for $C_{15}H_{33}N_4O_4$ $[M+H]^{+}$, 333.2503. Found 333.2495.

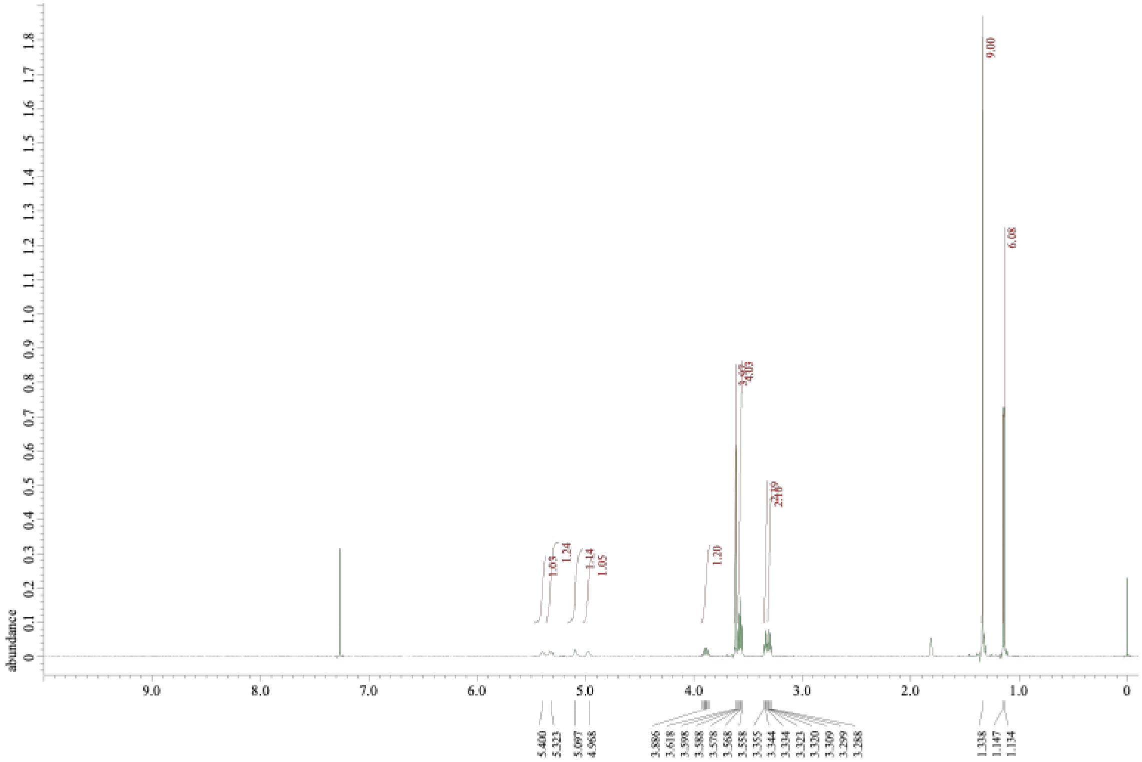

Fig. S15. ^{1}H NMR spectrum (500 MHz) of receptor **2c** in $CDCl_3$.

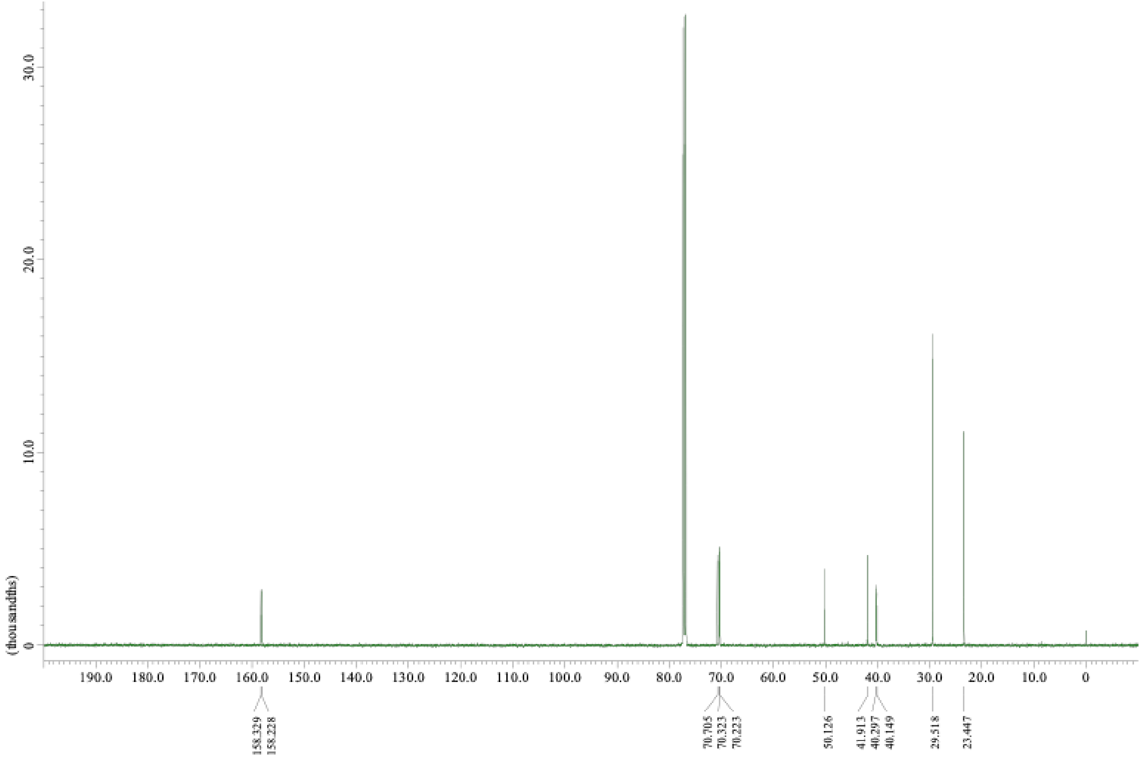

Fig. S16. ^{13}C NMR spectrum (126 MHz) of receptor **2c** in $CDCl_3$.

1-(2-(3-Butylureido)ethoxy)-2-(2-(3-*tert*-butylureido)ethoxy)ethane (2d)

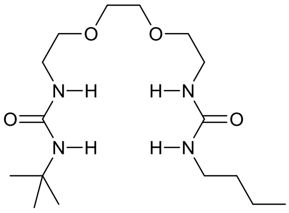

Yield 97%. M. p. 71.0–73.0 °C. ^{1}H NMR (500 MHz, $CDCl_3$) δ 3.62 (s, 4H), 3.58 (t, 2H, *J* = 4.9 Hz), 3.56 (t, 2H, *J* = 4.9 Hz), 3.35 (t, 2H, *J* = 5.0 Hz), 3.30 (t, 2H, *J* = 5.0 Hz), 3.17 (t, 2H, *J* = 7.0 Hz), 1.47 (quint, 2H, *J* = 0.00 Hz), 1.37 (m, 2H), 1.33 (s, 9H), 0.91(t, 3H, *J* = 7.5 Hz). ^{13}C NMR (126 MHz, $CDCl_3$) δ 159.1, 158.3, 70.7, 70.7, 70.3, 70.2, 50.1, 40.3, 40.1, 32.4, 29.5, 20.0, 13.8. HRMS (ESI^+): Calcd for $C_{16}H_{35}N_4O_4$ $[M+H]^+$, 347.2660. Found 347.2655.

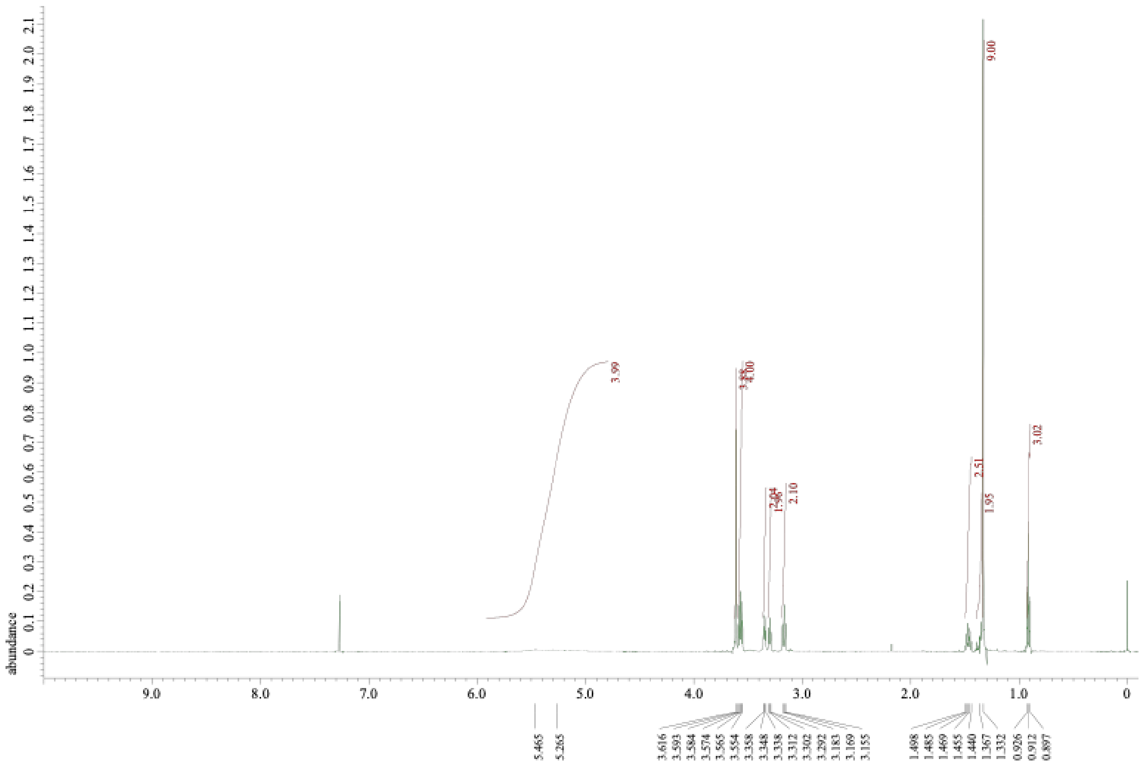

Fig. S17. ^{1}H NMR spectrum (500 MHz) of receptor **2d** in $CDCl_3$.

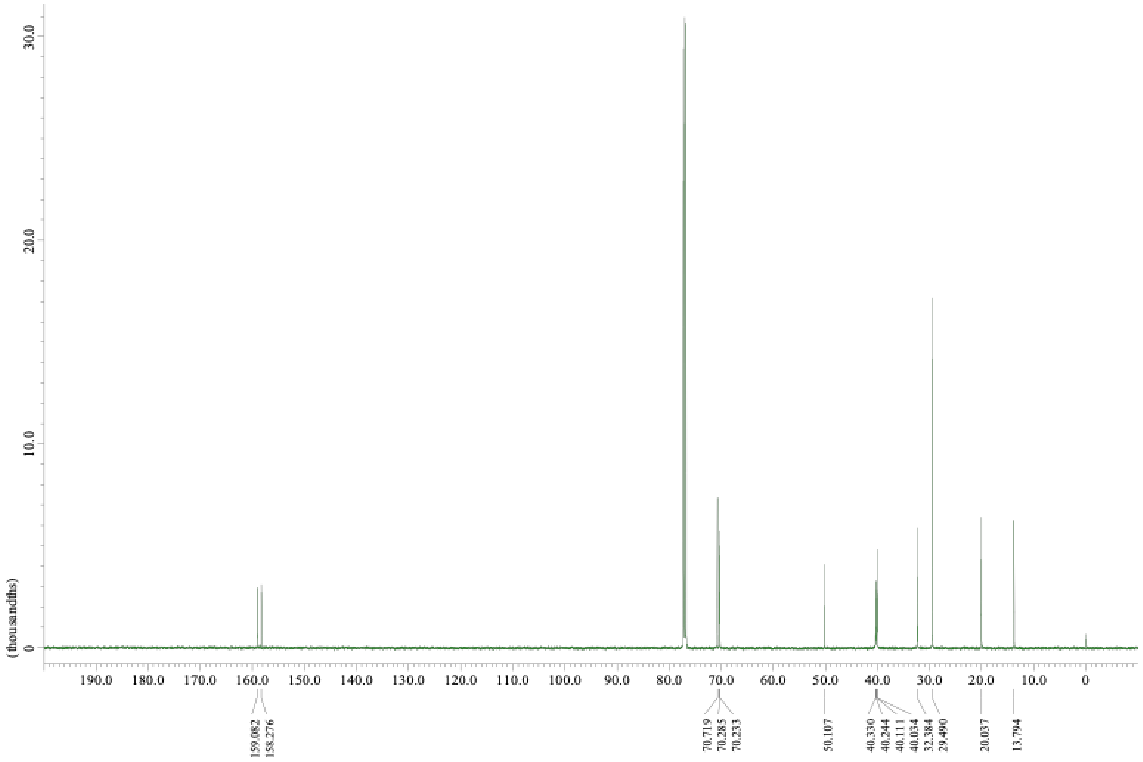

Fig. S18. ^{13}C NMR spectrum (126 MHz) of receptor **2d** in $CDCl_3$.

1-(2-(3-*tert*-Butylureido)ethoxy)-2-(2-(3-dodecylureido)ethoxy)ethane (2e)

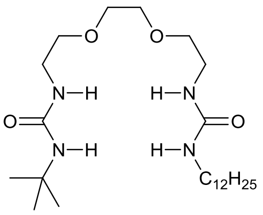

Yield 61%. M. p. 65.0–67.0 °C. ^{1}H NMR (500 MHz, $CDCl_3$) δ 5.36 (bs, 2H), 5.14 (bs, 2H), 3.62 (s, 4H), 3.59 (t, 2H, *J* = 5.1 Hz), 3.57 (t, 2H, *J* = 5.1 Hz), 3.35 (t, 2H, *J* = 5.0 Hz), 3.30 (t, 2H, *J* = 5.0 Hz), 3.16 (t, 2H, *J* = 7.2 Hz), 1.48 (quint, 2H, *J* = 7.2 Hz), 1.34 (s, 9H), 1.31-1.25(m, 18H), 0.88(t, 3H, *J* = 7.0 Hz). ^{13}C NMR (126 MHz, $CDCl_3$) δ 159.0, 158.3, 70.7, 70.3, 70.2, 50.1, 40.4, 40.4, 40.1, 31.9, 30.3, 29.7, 29.6, 29.6, 29.6, 29.5, 29.4, 29.3, 27.0, 22.7, 14.1. HRMS (ESI^+): Calcd for $C_{24}H_{51}N_4O_4$ $[M+H]^+$, 459.3913. Found 459.3905.

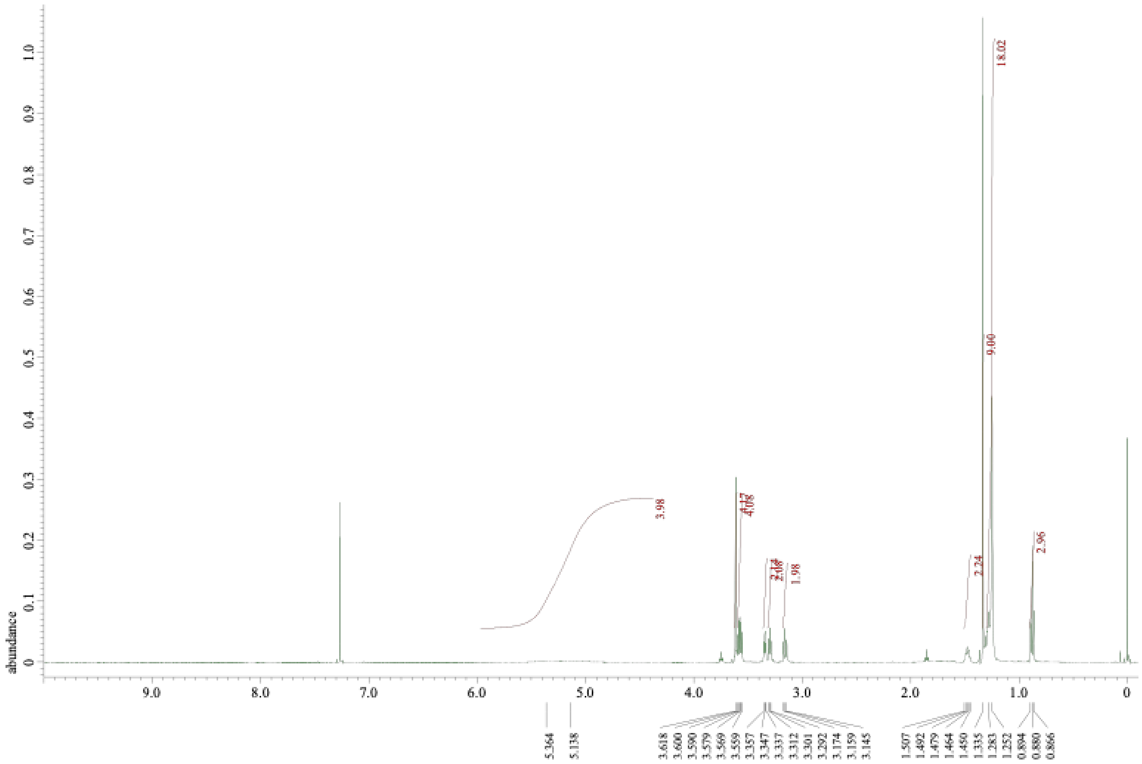

Fig. S19. ^{1}H NMR spectrum (500 MHz) of receptor **2e** in $CDCl_3$.

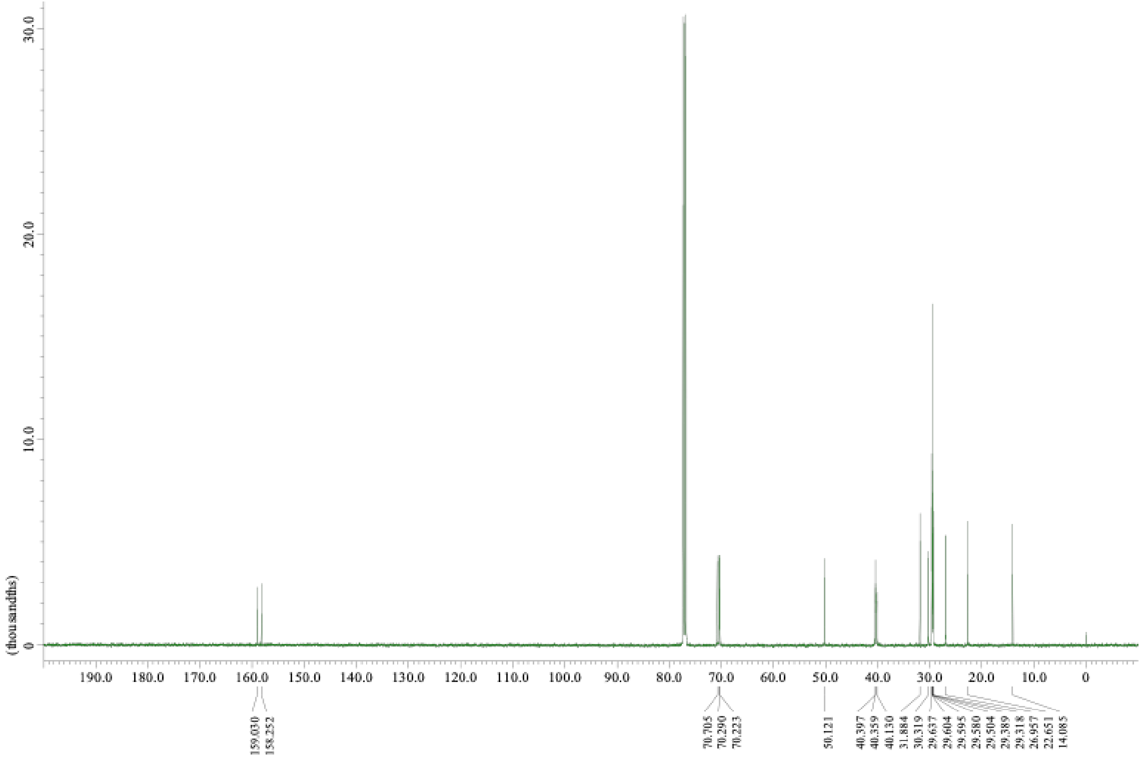

Fig. S20. ^{13}C NMR spectrum (126 MHz) of receptor **2e** in $CDCl_3$.

1-(2-(3-*tert*-Butylureido)ethoxy)-2-(2-(3-phenylureido)ethoxy)ethane (2f)

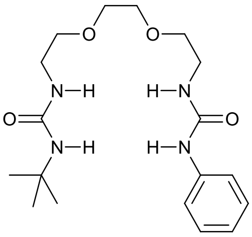

Yield 38%. M. p. 89.0–95.5 °C. ^{1}H NMR (500 MHz, $CDCl_3$) δ 8.05 (s, 1H), 7.43 (d, 2H, *J* = 8.5 Hz), 7.24 (t, 2H, *J* = 7.5, 8.5 Hz), 6.97 (t, 1H, *J* = 7.5 Hz), 6.13 (s, 1H), 5.11 (t, 1H, *J* = 5.3 Hz), 4.84 (s, 1H), 3.64-3.57 (m, 8H), 3.45 (q, 2H, *J* = 5.3 Hz), 3.25 (q, 2H, *J* = 5.3 Hz), 1.34 (s, 9H). ^{13}C NMR (126 MHz, $CDCl_3$) δ 158.4, 156.5, 139.7, 128.8, 122.2, 119.2, 70.8, 70.6, 70.3, 70.0, 50.4, 40.5, 39.9, 29.5. HRMS (ESI^+): Calcd for $C_{18}H_{31}N_4O_4$ $[M+H]^+$, 367.2347. Found 367.2343.

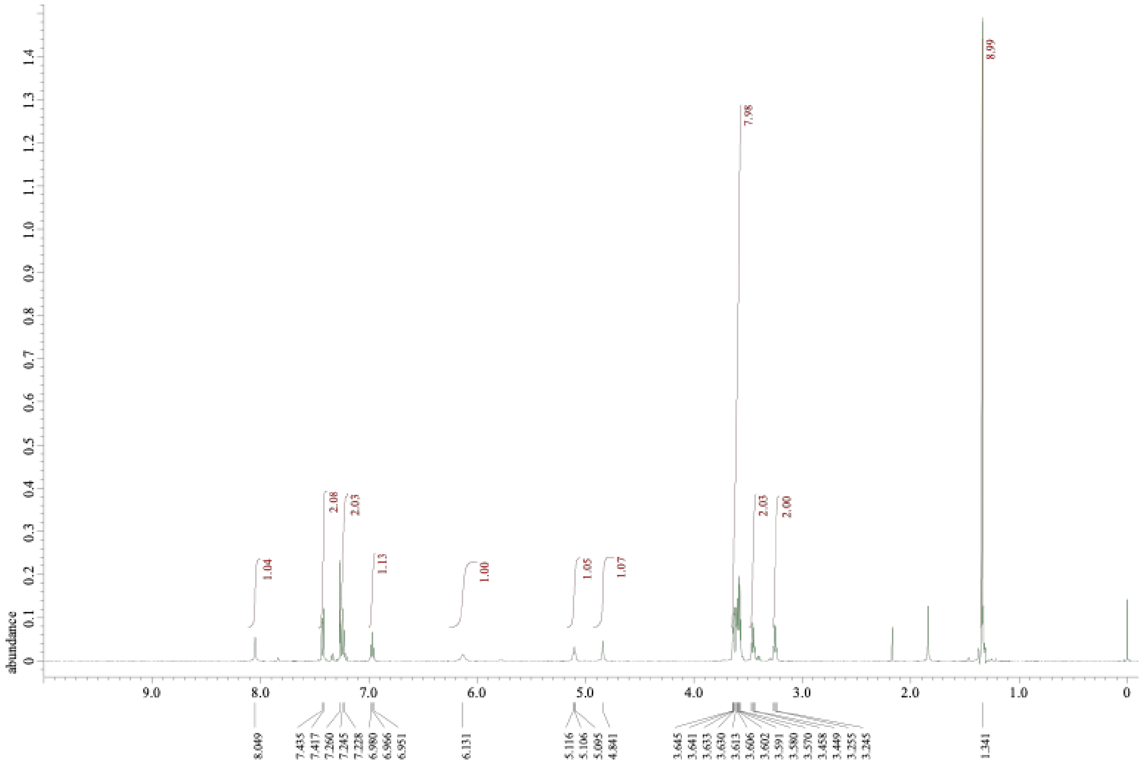

Fig. S21. ^{1}H NMR spectrum (500 MHz) of receptor **2f** in $CDCl_3$.

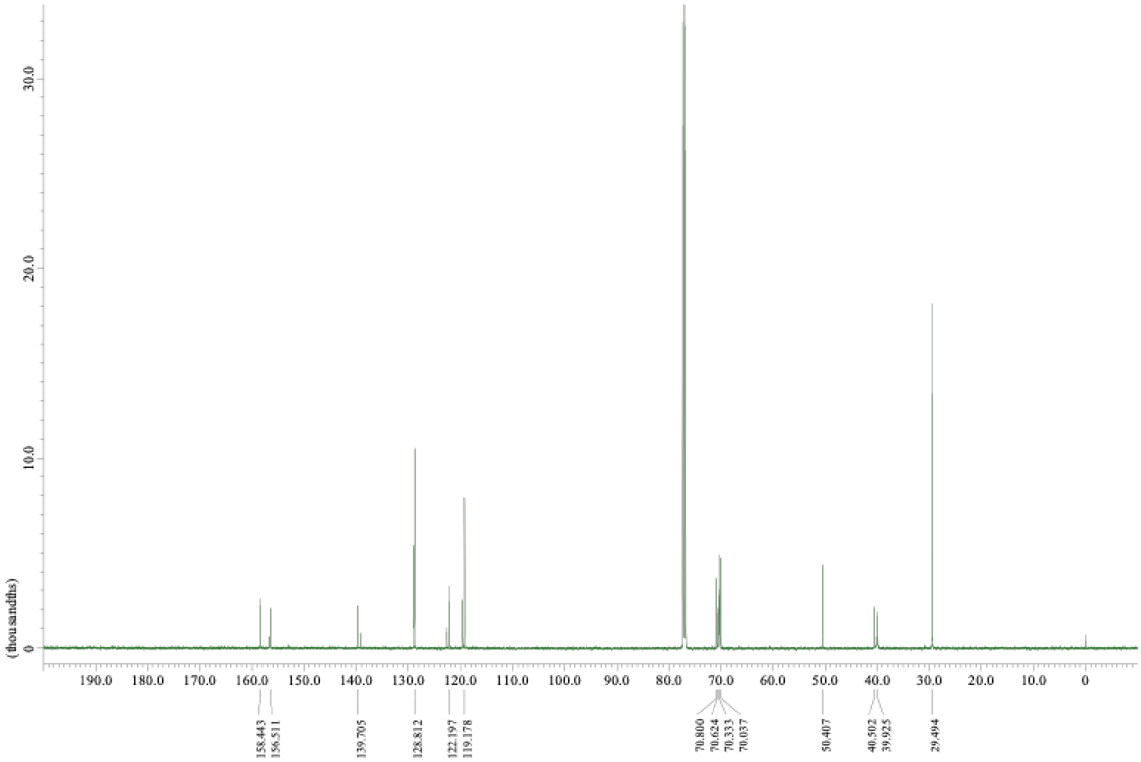

Fig. S22. ^{13}C NMR spectrum (126 MHz) of receptor **2f** in $CDCl_3$.

1-(2-(3-*tert*-Butylureido)ethoxy)-2-(2-(3-(4-trifluoromethylphenyl)ureido)ethoxy)ethane (2g)

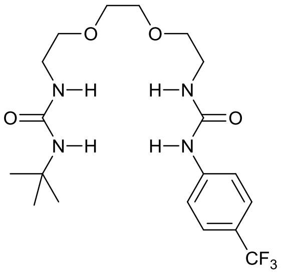

Yield 88%. M. p. 44.5–46.5 °C. ^{1}H NMR (500 MHz, $CDCl_3$) δ 8.63 (s,1H), 7.59 (d, 2H, J = 8.5 Hz), 7.48 (d, 2H, J = 8.5 Hz), 6.37 (s, 1H), 4.96 (s, 1H), 4.65 (s, 1H), 3.68-3.64 (m, 4H), 3.63-3.57 (m, 4H), 3.49 (q, 2H, J = 4.8 Hz), 3.23 (q, 2H, J = 4.9 Hz), 1.38 (s, 9H). ^{13}C NMR (126 MHz, $CDCl_3$) δ 158.6, 156.0, 143.4, 126.0 (q, $^{3}J_{CF}$ = 3.7 Hz), 124.5 (q, $^{1}J_{CF}$ = 272 Hz), 123.3 (q, $^{2}J_{CF}$ = 32 Hz), 117.8, 70.7, 70.4, 69.9, 69.8, 50.6, 40.9, 39.7, 29.5. HRMS (ESI^{+}): Calcd for $C_{19}H_{30}F_3N_4O_4$ $[M+H]^{+}$, 435.2220. Found 435.2223.

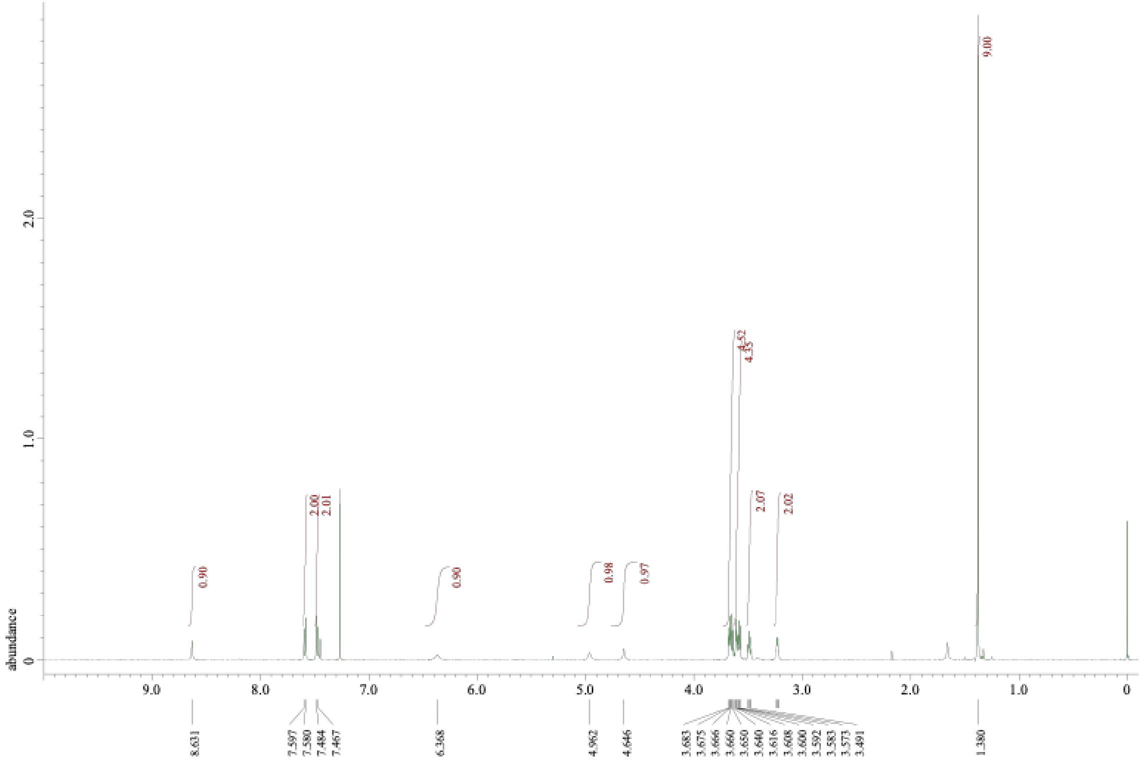

Fig. S23. ^{1}H NMR spectrum (500 MHz) of receptor **2g** in $CDCl_3$.

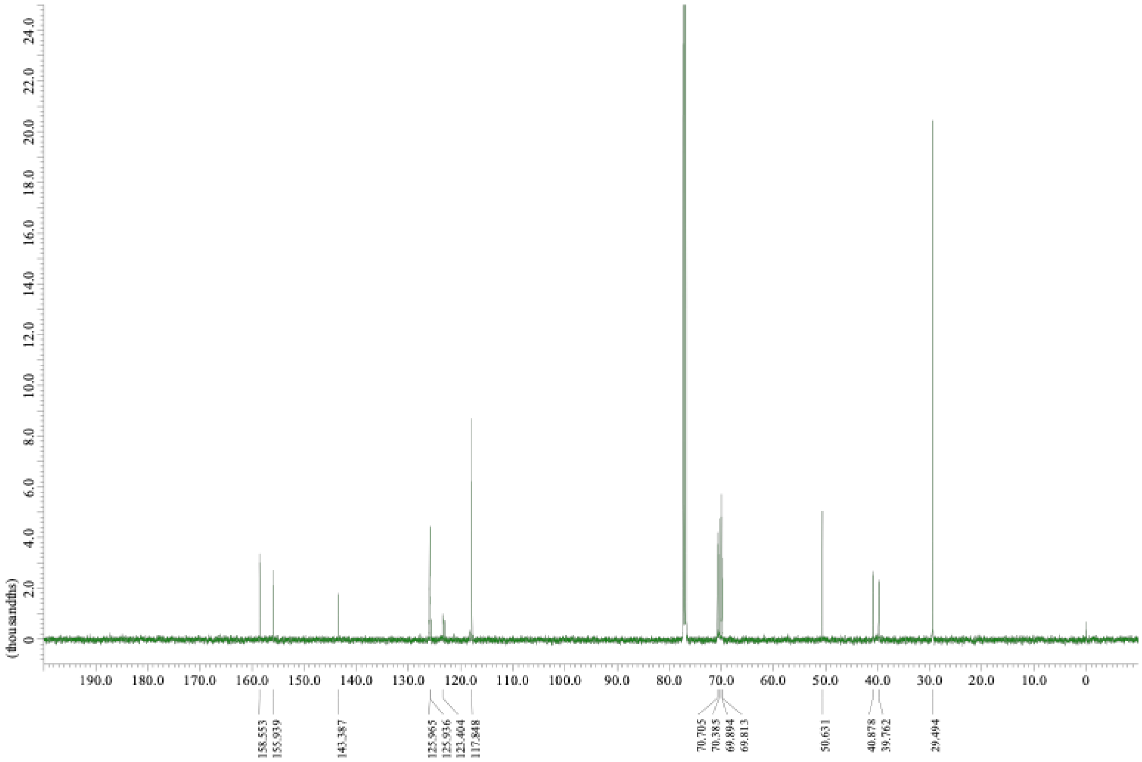

Fig. S24. ^{13}C NMR spectrum (126 MHz) of receptor **2g** in $CDCl_3$.

1-(2-(3-*tert*-Butylureido)ethoxy)-2-(2-(3-(4-nitrophenyl)ureido)ethoxy)ethane (2h)

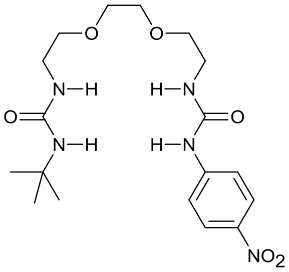

Yield 63%. M. p. 51.0–53.0 °C. ^{1}H NMR (500 MHz, $CDCl_3$) δ 9.06 (s, 1H), 8.13 (d, 2H, *J* = 9.3 Hz), 7.63 (d, 2H, *J* = 9.3 Hz), 6.47 (s, 1H), 4.93 (s, 1H), 4.64 (s, 1H), 3.69-3.65 (m, 4H), 3.62-3.56 (m, 4H), 3.50 (q, 2H, *J* = 5.4 Hz), 3.24 (q, 2H, *J* = 5.4 Hz), 1.39 (s, 9H). ^{13}C NMR (126 MHz, $CDCl_3$) δ 1586, 155.3, 146.8, 141.4, 125.1, 117.2, 70.5, 70.4, 69.8, 69.6, 50.8, 41.1, 39.7, 29.5. HRMS (ESI^+): Calcd for $C_{18}H_{30}N_5O_6$ $[M+H]^+$, 412.2197. Found 412.2200.

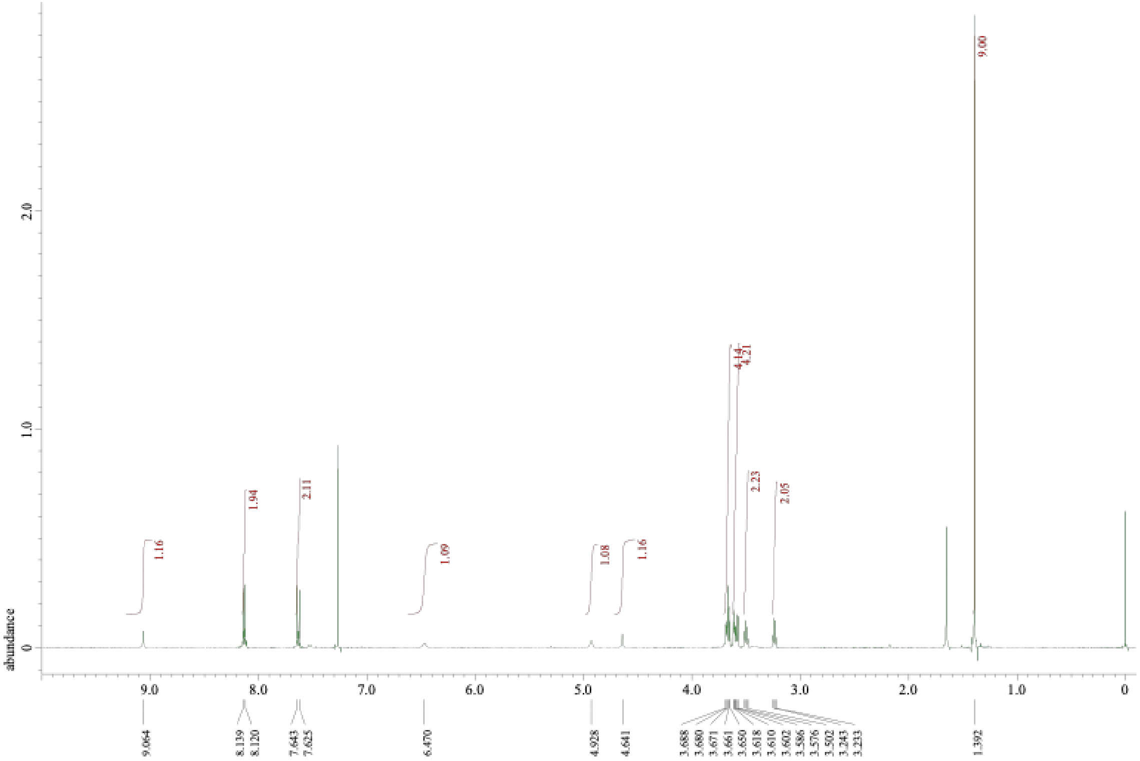

Fig. S25. ^{1}H NMR spectrum (500 MHz) of receptor **2h** in $CDCl_3$.

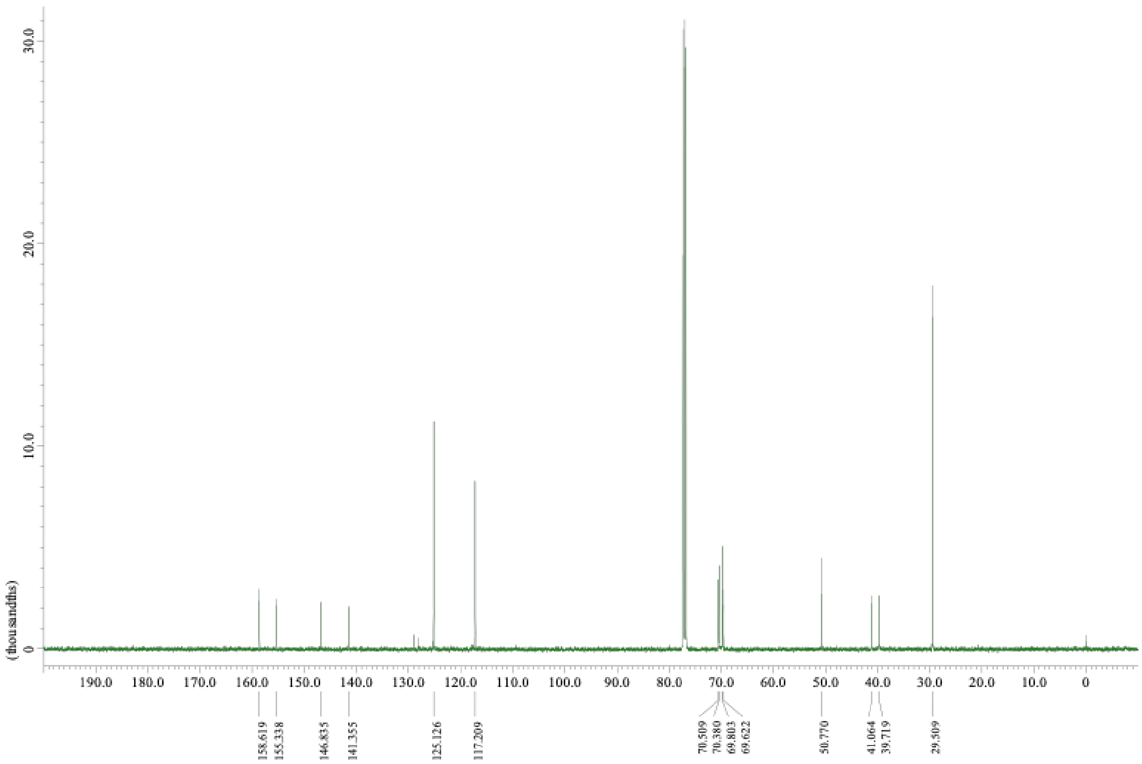

Fig. S26. ^{13}C NMR spectrum (126 MHz) of receptor **2h** in $CDCl_3$.

Saturated concentration of receptors

A saturated solution of receptor in $CDCl_3$ and MeCN-d_3 was prepared. An appropriate amount of the solution was added into an NMR tube via a microsyringe, and the solution was evaporated under reduced pressure. Into the NMR tube, 600 μL of naphthalene (2.0 mM) solution in $CDCl_3$ was added and the NMR was measured. From the integration of the receptor and naphthalene, the saturation concentration was determined.

NMR titrations of receptor with cation and anion

A solution of receptor was prepared ([receptor] = 10 mM in MeCN-d_3) and filled into an NNR tube, then the ^{1}H NMR was measured. An aliquot of stock solution of guest salts (TBACl, TBAAcO, $LiPF_6$, and $NaPF_6$) in MeCN-d_3 was added to the NMR tube, followed by ^{1}H NMR of the mixture was measured. The process was repeated to obtain the titration data. The association constants were calculated from the data by BindFit.[2-3] The titrations were performed at least in triplicate to ensure the accuracy of the results.

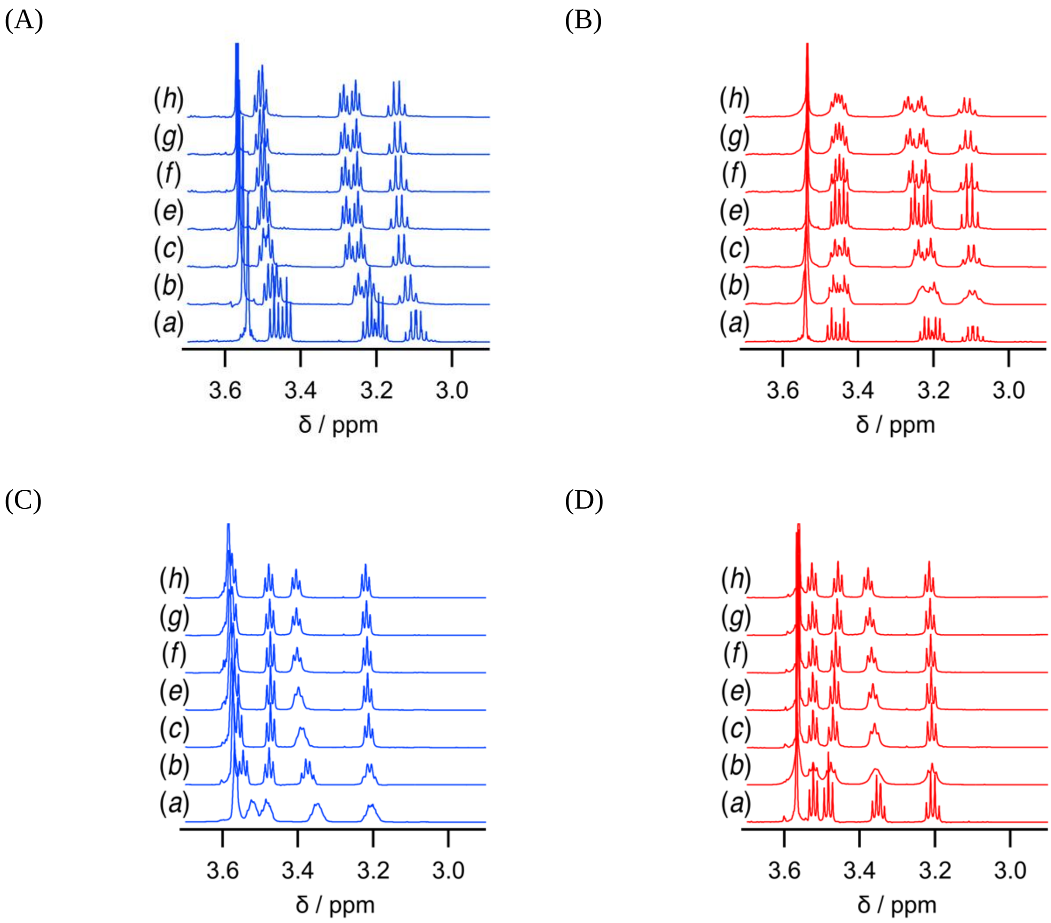

Fig. S27. ^{1}H NMR spectra of **2b** upon the addition of 0–4.0 eq of $LiPF_6$ (A) and $NaPF_6$ (B), and of **2g** upon the addition of 0–4.0 eq of $LiPF_6$ (C) and $NaPF_6$ (D) in MeCN-d_3 at 298 K.

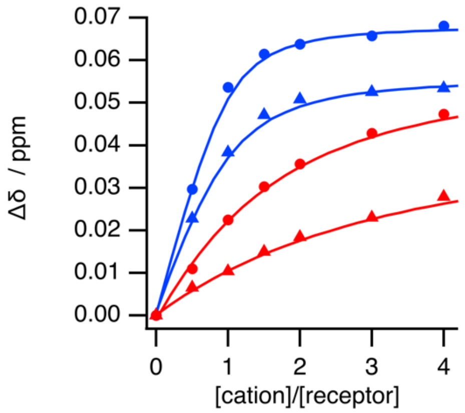

Fig. S28. Chemical shift changes of receptors **2b** (●) and **2g** (▲) ether methylene groups upon the addition of $LiPF_6$ (blue) and $NaPF_6$ (red) in MeCN-d_3 at 298 K. [Receptor] = 1.0×10^{-2} M.

UV-vis titrations of receptor with cation and anion

A solution of the receptor was prepared ([receptor] = 2.0×10^{-5} M) in MeCN and placed in a UV cuvette. Then the UV spectrum of the solution was measured. An aliquot of the stock solution of guest salts (TBACl and TBAAcO) in MeCN was added to the cuvette. Then the UV spectrum of the mixture was measured. This process was repeated to obtain the titration data. The association constants were calculated from the data by BindFit.[2-3] The titrations were performed in at least triplicate to ensure accuracy.

Table S1. The URLs of the saved data from the curve fitting analyses of the complexation of receptors and guests by BindFit.

Receptor	Guest	URL of the saved data
**1a**	TBAAcO	http://app.supramolecular.org/bindfit/view/58811a33-27c2-4506-b7e6-bebd568444d2
**1a**	TBACl	http://app.supramolecular.org/bindfit/view/2370284a-23cf-4c25-9a8c-85967c51c06e
**1b**	TBAAcO	http://app.supramolecular.org/bindfit/view/ddc9a7ae-c06c-43b7-9412-28880c747c35
**1b**	TBACl	http://app.supramolecular.org/bindfit/view/b8f2791b-7b7b-4834-afb8-44cce2a3ab82
**1d**	TBAAcO	http://app.supramolecular.org/bindfit/view/ec8a64d5-099f-4672-b728-70f410a56824
**1d**	TBACl	http://app.supramolecular.org/bindfit/view/c77f56a7-c42c-4dcc-afe1-e71a700b94e0
**1f**	TBAAcO	http://app.supramolecular.org/bindfit/view/40708ea3-c236-456a-863d-299e23bc5ee7
**1f**	TBACl	http://app.supramolecular.org/bindfit/view/40708ea3-c236-456a-863d-299e23bc5ee7
**1g**	TBAAcO	http://app.supramolecular.org/bindfit/view/e70cf70d-58ab-47aa-af55-433129a24004
**1g**	TBACl	http://app.supramolecular.org/bindfit/view/a482e10c-c021-4427-a938-2b2c6c9341e2
**1h**	TBAAcO	http://app.supramolecular.org/bindfit/view/8bd0aad3-4b04-4cbb-a63f-680e7a04370c
**1h**	TBACl	http://app.supramolecular.org/bindfit/view/1636fd0e-8934-4ae5-9136-322b36a

		0252e
**2b**	$LiPF_6$	http://app.supramolecular.org/bindfit/view/d5ceaf75-0bf7-4f78-b5fb-4c1ea5ae3608
**2b**	$NaPF_6$	http://app.supramolecular.org/bindfit/view/b4ec8418-92dd-47e7-90df-c23fc65ce1af
**2b**	TBAAcO	http://app.supramolecular.org/bindfit/view/2d5eee62-bcab-4ea7-be0c-5adf9873df61
**2b**	TBACl	http://app.supramolecular.org/bindfit/view/e0713adc-b338-483e-8d24-8068d3469ee7
**2d**	TBAAcO	http://app.supramolecular.org/bindfit/view/3611a75f-ab2f-40ae-bdf1-b5c55df8797b
**2d**	TBACl	http://app.supramolecular.org/bindfit/view/3611a75f-ab2f-40ae-bdf1-b5c55df8797b
**2f**	TBAAcO	http://app.supramolecular.org/bindfit/view/f003a300-6618-4951-b05f-a141fb468192
**2f**	TBACl	http://app.supramolecular.org/bindfit/view/48252453-a2d9-4c49-9255-9f811e7fd368
**2g**	$LiPF_6$	http://app.supramolecular.org/bindfit/view/39316e14-3562-42a6-94c5-08183d9addac
**2g**	$NaPF_6$	http://app.supramolecular.org/bindfit/view/8f1adfc1-e8e3-4134-a1f9-91c49f4032a6
**2g**	TBAAcO	http://app.supramolecular.org/bindfit/view/e0b05d6f-66f6-429c-8767-e7528ca87074
**2g**	TBACl	http://app.supramolecular.org/bindfit/view/bffc4ae0-ef5e-4d30-b523-e9fc655f8a7e
**2h**	TBAAcO	http://app.supramolecular.org/bindfit/view/e1a92a67-2344-40a7-971f-c6f51c03abc1
**2h**	TBACl	http://app.supramolecular.org/bindfit/view/eb31e30c-5134-4fb7-8542-41ef9b9b92b5

Solid-liquid extraction of inorganic salts with receptors in $CDCl_3$

A solution of receptor was prepared ([receptor] = 10 mM in $CDCl_3$). Into an NMR tube, 500 μl of the solution was added, and the NMR was measured. Finely powdered solid salt (100 eq.) was added into the NMR tube, the mixture was stirred vigorously at room temperature, and the NMR was measured.

Measurement of viscosity

The viscosity of acetonitrile solutions of **1a**·LiCl, **2b**·LiCl, and **2g**·LiCl (0.25, 1.0, and 3.0 M) was measured with a viscometer (Viscometer TV-22, Toki Sangyo Co., Ltd, Japan). The viscosity of 0.025 M LiCl solutions was also measured. A sample (1 mL) was loaded, the viscosity was obtained at a rotational speed of 50 rpm under 25 °C after standing for 2 min to stabilize the temperature. The results are summarized in Table 3.

Measurement of ionic conductivity

A solution (3 M) of receptor and LiCl in MeCN was placed in a two-terminal cell and the impedance was measured. The impedance of the solution was calculated from the resistance determined from the Cole-Cole plot and the cell constant (0.2826 cm^{-1}).

DFT calculations

The optimized structures of **2b**·LiCl and **2g**·LiCl were performed by Gaussian 16, Revision A.03 [4] at the B3LYP-D3/6-31+G(d) level of theory in chloroform (PCM). The energetically lowest structures are shown in Figure 7, and the Cartesian coordinates of the structure are shown in Tables S2 and S3, respectively.

Table S2. Cartesian coordinates of the optimized structure of **2b**·LiCl complex in chloroform by DFT calculation (B3LYP-D3/6-31+G(d) level of theory in chloroform (PCM)).

Atom	X	Y	Z	Atom	X	Y	Z
C	-2.162	-3.369	-0.51	C	3.403	0.727	0.022
C	-3.164	-2.583	0.316	C	4.894	0.431	-0.215
H	-1.985	-4.347	-0.044	C	2.713	1.132	-1.296
H	-2.538	-3.52	-1.531	C	3.266	1.861	1.052
O	-0.952	-2.605	-0.561	H	2.213	2.08	1.266
H	-4.16	-3.039	0.231	H	3.756	1.593	1.995
H	-2.869	-2.558	1.375	H	3.732	2.775	0.668
O	-3.168	-1.253	-0.209	H	5.375	0.131	0.724
C	-4.122	-0.362	0.381	H	5.019	-0.373	-0.943
H	-5.141	-0.676	0.121	H	5.396	1.331	-0.589
H	-4.017	-0.385	1.477	H	2.792	0.326	-2.031
C	-3.834	1.029	-0.168	H	1.652	1.349	-1.122
N	-2.445	1.375	0.131	H	3.181	2.031	-1.715
H	-3.96	1.04	-1.253	H	-2.191	1.29	1.113
H	-4.545	1.747	0.264	C	-1.816	2.417	-0.555
C	0.173	-3.278	-1.167	O	-2.305	2.936	-1.564
H	-0.178	-4.154	-1.726	N	-0.586	2.745	-0.055
H	0.629	-2.577	-1.871	H	-0.269	2.276	0.788
C	1.185	-3.676	-0.095	C	0.165	3.901	-0.536
N	1.703	-2.526	0.632	H	0.007	3.973	-1.616
H	0.717	-4.351	0.63	C	-0.239	5.209	0.152
H	2.017	-4.205	-0.567	H	1.226	3.692	-0.372
H	1.055	-2.107	1.294	H	-1.298	5.425	-0.027
C	2.574	-1.658	-0.009	H	-0.075	5.148	1.234
O	3.181	-1.99	-1.04	H	0.354	6.045	-0.239
N	2.729	-0.448	0.62	Li	-1.228	-0.657	-0.104
H	2.009	-0.222	1.302	Cl	-0.37	-0.188	2.056

Table S3. Cartesian coordinates of the optimized structure of **2g**·LiCl complex in chloroform by DFT calculation (B3LYP-D3/6-31+G(d) level of theory in chloroform (PCM)).

Atom	X	Y	Z	Atom	X	Y	Z
C	-5.901	-1.14	-0.11	H	1.013	1.108	0.402
C	-5.076	-2.407	-0.2	H	1.168	2.845	0.051
H	-6.921	-1.319	-0.48	H	1.571	2.214	1.663
H	-5.946	-0.801	0.93	H	-0.452	4.503	1.079
O	-5.254	-0.142	-0.91	H	-1.723	3.95	2.193
H	-5.511	-3.165	0.461	H	-0.017	3.893	2.691
H	-5.056	-2.807	-1.23	H	-1.982	1.417	2.657
O	-3.74	-2.08	0.217	H	-0.819	0.275	1.937
C	-3.001	-3.21	0.719	H	-0.296	1.38	3.222
H	-3.625	-3.74	1.451	H	-1.105	-1.808	-0.405
H	-2.765	-3.895	-0.11	C	0.537	-2.025	0.811
C	-1.739	-2.733	1.414	O	1.001	-2.362	1.906
N	-0.769	-2.222	0.463	N	1.278	-1.423	-0.201
H	-2.004	-1.974	2.164	H	0.755	-1.163	-1.036
H	-1.295	-3.579	1.949	C	2.621	-1.042	-0.192
C	-5.688	1.201	-0.64	Li	-3.284	-0.423	-1.073
H	-6.729	1.331	-0.96	Cl	-1.291	-0.491	-2.272
H	-5.618	1.389	0.441	C	3.497	-1.255	0.889
C	-4.771	2.151	-1.4	C	3.107	-0.394	-1.348
N	-3.359	1.842	-1.14	C	4.427	0.031	-1.423
H	-4.929	2.037	-2.47	C	4.82	-0.825	0.804
H	-5.018	3.181	-1.12	C	5.295	-0.185	-0.345
H	-2.761	1.902	-1.96	H	2.44	-0.225	-2.189
C	-2.77	2.294	0.058	H	4.781	0.526	-2.321
O	-3.464	2.715	0.988	H	3.138	-1.75	1.78
N	-1.411	2.176	0.076	H	5.485	-0.996	1.645
H	-1.008	1.604	-0.66	C	6.7	0.325	-0.405
C	-0.562	2.339	1.284	F	7.221	0.276	-1.662
C	-0.704	3.762	1.847	F	6.8	1.635	-0.015
C	-0.942	1.286	2.342	F	7.548	-0.368	0.401
C	0.884	2.113	0.816				

References

[1] T. Mimuro, A. Yoshida, K. Kamo, M. Hirasawa, S. Kondo, *Org. Biomol. Chem.* **2023**, *21*, 5281-5287.

[2] http://supramolecular.org

[3] D. B. Hibbert, P. Thordarson, *Chem. Commun.* **2016**, *52*, 12792-12805.

[4] M. J. T. Frisch, G. W.; Schlegel, H. B.; Scuseria, G. E.; Robb, M. A.; Cheeseman, J. R.; Scalmani, G.; Barone, V.; Petersson, G. A.; Nakatsuji, H.; Li, X.; Caricato, M.; Marenich, A. V.; Bloino, J.; Janesko, B. G.; Gomperts, R.; Mennucci, B.; Hratchian, H. P.; Ortiz, J. V.; Izmaylov, A. F.; Sonnenberg, J. L.; Williams-Young, D.; Ding, F.; Lipparini, F.; Egidi, F.; Goings, J.; Peng, B.; Petrone, A.; Henderson, T.; Ranasinghe, D.; Zakrzewski, V. G.; Gao, J.; Rega, N.; Zheng, G.; Liang, W.; Hada, M.; Ehara, M.; Toyota, K.; Fukuda, R.; Hasegawa, J.; Ishida, M.; Nakajima, T.; Honda, Y.; Kitao, O.; Nakai, H.; Vreven, T.; Throssell, K.; J. A. Montgomery, J.; Peralta, J. E.; Ogliaro, F.; Bearpark, M. J.; Heyd, J. J.; Brothers, E. N.; Kudin, K. N.; Staroverov, V. N.; Keith, T. A.; Kobayashi, R.; Normand, J.; Raghavachari, K.; Rendell, A. P.; Burant, J. C.; Iyengar, S. S.; Tomasi, J.; Cossi, M.; Millam, J. M.; Klene, M.; Adamo, C.; Cammi, R.; Ochterski, J. W.; Martin, R. L.; Morokuma, K.; Farkas, O.; Foresman, J. B.; Fox, D. J., Gaussian, Inc., Wallingford CT, **2016**.
